# Disproportionality Analysis of Fluoroquinolone‐Associated Peripheral Neuropathy in the FAERS Database (2007–2024)

**DOI:** 10.1111/cts.70541

**Published:** 2026-04-14

**Authors:** Nimra Shamim, Kelly Doughty, Hau‐Tak Chau, James Brown, Robert Baldock, Ngan Pan Bennett Au

**Affiliations:** ^1^ School of Medicine, Pharmacy and Biomedical Sciences, Faculty of Science and Health University of Portsmouth Portsmouth UK; ^2^ Institute of Life Sciences and Healthcare University of Portsmouth Portsmouth UK; ^3^ Department of Medicine, School of Clinical Medicine The University of Hong Kong Hong Kong SAR Hong Kong; ^4^ Department of Comparative Biomedical Sciences, School of Veterinary Medicine University of Surrey Guildford UK

**Keywords:** adverse events/reaction, ciprofloxacin, delafloxacin, fluoroquinolone, gemifloxacin, levofloxacin, moxifloxacin, ofloxacin, peripheral neuropathy, pharmacovigilance

## Abstract

Fluoroquinolones (FQs) are among the most frequently prescribed antibiotic classes worldwide. Despite their therapeutic versatility in treating bacterial infections, regulatory authorities recognized risks of persistent and potentially irreversible adverse effects, particularly peripheral neuropathy (PN). However, a comprehensive pharmacovigilance assessment of PN‐related adverse events (AEs) across all six FDA‐approved FQs remains limited. We therefore analyzed adverse event reports (AERs) associated with these six agents from the FDA Adverse Event Reporting System (FAERS) Public Dashboard spanning 2007–2024 Q3, following READUS‐PV guidelines. Disproportionality analysis was conducted to identify potential safety signals for PN‐related AEs reported with FQ use following deduplication and exclusion of cases involving non‐FQ concomitant medications. Positive disproportionality signals were observed for the commonly prescribed FQs (ciprofloxacin, levofloxacin, moxifloxacin, and ofloxacin), with seven distinct PN manifestations generating signals. Notably, we detected PN‐related signals for gemifloxacin, a relatively new FQ with limited prior evaluation of neurotoxicity. Univariate logistic regression revealed that women and adults aged 18–64 years were more frequently represented in FQ‐associated PN‐related AERs, whereas men and patients aged ≥ 65 years were disproportionately represented among cases with fatal outcomes. Combination therapy with multiple FQs generated disproportionality signals exceeding those with FQ monotherapy. These findings underscore the need for increased vigilance when prescribing FQs, particularly for mild infections where risks may outweigh benefits. Strengthened clinical monitoring for early signs of PN is advisable when FQ treatment becomes unavoidable. Further controlled epidemiological studies are needed to validate these signals and define at‐risk groups, alongside mechanistic research aimed at supporting the development of neuroprotective strategies.

## Study Highlights

- What is the current knowledge on the topic?
  ○ Fluoroquinolone (FQ) antibiotics remain widely prescribed globally, with growing concerns regarding severe adverse drug reactions, including peripheral neuropathy (PN). Regulatory authorities have reviewed FQ safety profiles and issued prescribing restrictions to limit use in uncomplicated infections; however, systematic signal analyses of FQ‐associated PN remain limited.
- What question did this study address?
  ○ Have reporting patterns of FQ‐associated adverse event reports (AERs) changed following the issue of boxed warnings in 2016? Are PN‐related adverse events (AEs) observed across all six FDA‐approved FQs? Which PN‐related AEs are most frequently reported with FQs? What factors may influence reporting of FQ‐associated PN‐related AEs?
- What does this study add to our knowledge?
  ○ PN‐related AEs were frequently reported among neurological events in the FAERS dataset. Disproportionality analysis detected safety signals for seven PN‐related AEs (paraesthesia, peripheral neuropathy, hypoaesthesia, burning sensation, sensory disturbance, small fiber neuropathy and hyperaesthesia), with gemifloxacin showing a signal for burning sensation. Combination FQ therapy displayed a reporting pattern suggesting higher odds of PN‐related AEs compared to monotherapy, warranting further investigation. Sex‐based differences in reporting were observed: PN was more frequently reported in females, whereas mortality was more commonly reported in males.
- How might this change clinical pharmacology or translational science?
  ○ These reporting patterns suggest the need for enhanced monitoring of FQ‐associated PN risk, particularly with combination therapy approaches. Our findings support the development of targeted adverse event screening protocols and improved post‐marketing surveillance strategies for FQ use in vulnerable populations. Further investigation into mechanistic pathways underlying PN pathogenesis and associated clinical factors is warranted to inform risk mitigation strategies.

## 1 Introduction

Fluoroquinolones (FQs) are broad‐spectrum antibiotics commonly prescribed for bacterial infections globally. Six FQs (ciprofloxacin, levofloxacin, moxifloxacin, ofloxacin, gemifloxacin, and delafloxacin) are approved by the US Food and Drug Administration (FDA), whereas five (except gemifloxacin) are authorized by the European Medicines Agency (EMA) and UK Medicines and Healthcare products Regulatory Agency (MHRA) (Table S1). By inhibiting DNA gyrase and topoisomerase IV [1], FQs remain important anti‐microbial agents, accounting for more than 10 million annual prescriptions in the US [2], with increasing use in low‐ and middle‐income countries [3].

Concerns have grown regarding serious adverse effects associated with FQ treatment, particularly peripheral neuropathy (PN) [4], a potentially irreversible condition. A UK cross‐sectional study demonstrated a 47% elevated PN risk following FQ exposure [5], prompting regulatory agencies to issue strengthened warnings (FDA: 2016, EMA: 2018, MHRA: 2024) [4]. Nevertheless, the extent to which these regulatory interventions have influenced reporting behaviors or prescribing patterns remains unclear.

This study evaluated the reporting of PN‐related adverse events (AEs) associated with all six FDA‐approved FQs, including recently introduced agents (gemifloxacin and delafloxacin), and assessed whether the 2016 FDA boxed warning altered reporting patterns. Disproportionality analysis of global real‐world data from the FDA Adverse Event Reporting System (FAERS) offers an efficient, scalable means to explore drug‐event associations and assess temporal trends following regulatory actions [6]. This approach enables systematic detection of disproportionality signals for PN‐related AEs across all FDA‐approved FQs that may inform clinical awareness of these potentially irreversible adverse effects and influence prescribing practices.

## 2 Methods

### 2.1 Ethics Statement

As FAERS contains anonymized and de‐identified patient records, ethical clearance and informed consent were exempted.

### 2.2 Source of Data

Pharmacovigilance analyses of PN‐related AEs associated with FQ treatment were conducted using FAERS Public Dashboard, an interactive web‐based tool enabling extraction of case reports for six FDA‐approved FQs (see Table S2), following READUS‐PV guidelines and FAERS usage guidance [6, 7]. FAERS contains self‐reported adverse event reports (AERs) submitted by consumers, healthcare professionals, and pharmaceutical companies, including patient demographics, medication use, adverse events, clinical indications, outcomes, and reporter characteristics. Adverse drug reactions were coded using preferred terms (PTs) from MedDRA version 27.1. From all FQ‐associated AERs, 531 neurological PTs were identified under the primary System Organ Class (SOC) ‘Nervous System Disorders’; 28 PTs clinically relevant to PN were manually selected for analysis (Table S9).

### 2.3 Data Processing and Descriptive Analysis

AERs were extracted on 16th January 2025, deduplicated by identifying reports with identical age, sex, country, initial FDA received date, drug used, clinical indications and adverse reactions, following WHO Uppsala Monitoring Centre guidelines [8]. AERs involving concomitant non‐FQ drugs were excluded. The final dataset comprised 37,778 cases in which an FDA‐approved FQ was the sole suspected drug (Figure S1). Descriptive analyses were conducted for all FQ‐associated AERs and PN‐specific cases, including age, sex, age group, reporter type, country, seriousness and clinical outcomes. Serious AEs included life‐threatening events, hospitalization, disability and death. Between‐group differences in categorical variables were assessed using Chi‐square test or Fisher's exact test.

### 2.4 Signal Mining

For signal detection, Bayesian confidence propagation neural networks (BCPNN) were used to compute information components (ICs), with the full FAERS dataset (2007–2024 Q3) as comparator. Contingency tables were constructed and ICs were calculated using the faers R package. A positive signal was defined as IC > 2 with the lower 95% confidence interval (CI) > 0 [9, 10].

### 2.5 Time‐To‐Onset Analysis

Time‐to‐onset was calculated as the interval between treatment initiation (i.e., START_DT) and AE onset (i.e., EVENT_DT). Cumulative distribution curves were generated to characterize PN‐related time‐to‐onset patterns following FQ treatment across subgroups [10].

### 2.6 Statistical Analysis

Odds ratios (OR) for FQ‐associated PN‐related AEs were estimated using univariate logistic regression analysis. Missing age [11,406/37,778 (30.2%)] and sex [3908/37,778 (10.3%)] data were imputed using k‐nearest neighbors (kNN) algorithm with Euclidean distance across all available variables to identify nearest neighbors (*k* = 5) [10]. *p*‐Values were adjusted for FQ type, age, sex and clinical outcomes, with Bonferroni correction applied for multiple comparisons. All analyses were conducted using R (version 4.43) with ggplot2 for visualization. A two‐sided *p*‐value < 0.05 was considered statistically significant.

## 3 Results

### 3.1 An Overview of Fluoroquinolone (FQ)‐Associated Peripheral Neuropathy (PN) Adverse Events (AEs)

We investigated reporting patterns of PN‐related AEs among patients treated with six FDA‐approved FQs using publicly available FAERs database from 2007 (9 years before issuing the boxed warning) to 2024 Q3 (Figure S1). Following WHO Uppsala Monitoring Centre guidelines [8], duplicated reports and cases with concomitant non‐FQ drugs were excluded, identifying a total of 37,778 AERs over 17 years for descriptive analysis. As FAERS is a spontaneous reporting system where the total number of patients exposed to FQs remains unknown, the following analyses characterized reporting patterns and generated disproportionality signals rather than establishing causation or estimating true incidence rates.

**FIGURE 1:**
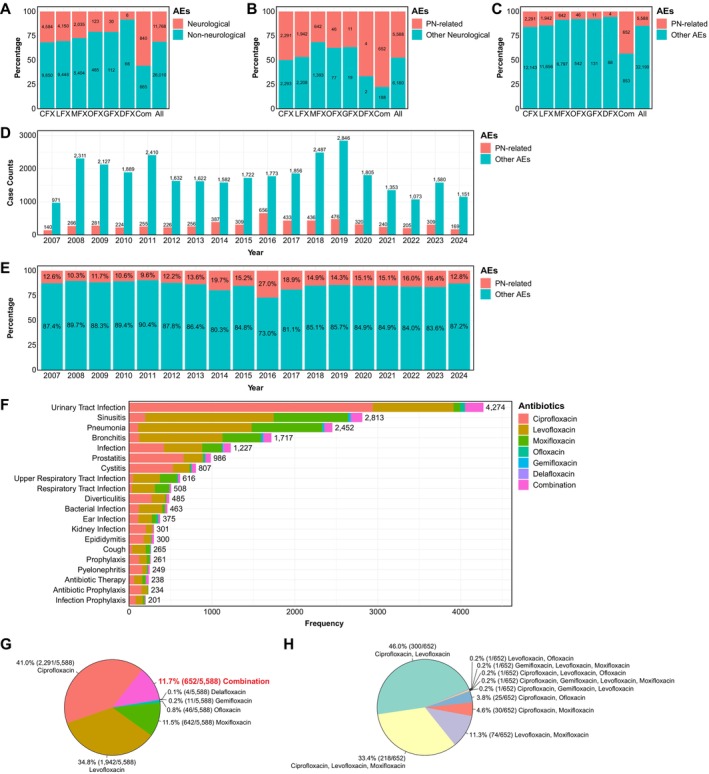
An overview of the clinical characteristic of FQ‐associated adverse event reports (AERs). (A) Of all 37,778 FQ‐associated AERs, 11,768 cases (31.2%) exhibited neurological AEs (CFX, ciprofloxacin; Com, combination therapy; DFX, delafloxacin; GFX, gemifloxacin; LFX, levofloxacin; MFX, moxifloxacin; OFX, ofloxacin). (B) Among these 11,768 cases with neurological AEs, 5588 AERs (47.5%) manifested PN‐related symptoms. (C) Overall, PN‐related AEs were identified in 5588 cases (14.8% of total FQ‐associated AERs). (D) Bar chart depicting the annual incident of FQ‐associated AERs with and without PN‐related AEs in the FAERS database between 2007 and 2024 Q3. (E) Proportional bar plot showing the relative distribution of FQ‐associated AERs with and without PN‐related AEs during the same period. (F) Clinical indications for FQ usage are presented in descending order of frequency, with bacterial infections constituting the majority of prescribing patterns. (G) Within FQ‐associated AERs with PN‐related AEs, ciprofloxacin was the most frequently implicated FQ, while combination therapy accounted for a notable proportion of the total cases. (H) The most common combination therapy of FQ treatment involved ciprofloxacin and levofloxacin, with or without moxifloxacin.

Within this reporting dataset, neurological AEs were frequently documented (11,768/37,778) (Figure 1A), with PN‐related AEs representing a substantial portion of neurological events (Figure 1B,C; Table S3). In 2016, FDA revised the Boxed Warning for FQs, highlighting risks of disabling and potentially permanent serious side effects, including peripheral neuropathy [4]. Temporal analysis using negative binomial regression revealed distinct patterns for total FQ‐associated AERs versus those with PN‐related AEs (Figure 1D). Total FQ‐associated AERs remained relatively stable during 2007–2015 (*p* = 0.8669), followed by a gradual increase from 2016 to 2019 (*p* = 0.0436) and a subsequent decline thereafter (*p* = 0.0070). In contrast, FQ‐associated AERs with PN‐related AEs increased gradually during 2007–2015 (*p* = 0.0075), a pronounced increase following the 2016 warning (*p* = 0.0034), then declined progressively (*p* = 1.46 × 10^−9^) (Figure 1D). PN‐related AEs accounted for a median of 14.62% of all FQ‐associated AERs [interquartile range (IQR): 12.27%–15.83%], with this proportion trending upward during 2007–2015 (*p* = 0.0758), rising non‐significantly after the 2016 warning (*p* = 0.1458), then declining significantly post‐2016 (*p* = 0.0058) (Figure 1E). While bacterial infections remain the primary indication for FQ use, we identified potential off‐label use, including cough (*N* = 265) (Figure 1F; Table S4). Female patients were more frequently represented in reports compared with males across different FQs (Figure S2B). Notably, most patients (75.19%) experienced serious outcomes (Figure S2E), with 898 cases (2.38% of total AERs) resulting in fatality (Figure S2F; Table S5).

Overall, we identified 5588 cases of PN‐related AEs associated with FQ use (Table S6). The median age of patients experiencing PN‐related AEs was 47 years (IQR: 36–57), with patients aged 35–64 years accounting for half of cases (2795/5588) (Figure S3A). PN‐related AERs were more frequently reported in females (3168/5588) than males (1988/5588) (Figure S3B). Most PN‐related AEs (3675/5588) were reported by consumers, with the United States as the primary reporting country (Figure S3C,D). Ciprofloxacin, the most widely prescribed FQ, accounted for the highest number of PN‐related AERs (2291/5588). Delafloxacin, despite FDA approval in 2017, was associated with four PN‐related AEs within 7 years. Combination FQ therapy contributed substantially to PN‐related AERs (652/5588) (Figure 1G), with ciprofloxacin‐levofloxacin (300/652) and ciprofloxacin‐levofloxacin‐moxifloxacin (218/652) being the most common combinations (Figure 1H). While most PN‐related AERs were classified as serious (Figure S3E), only 25 cases had fatal outcomes (Figure S3F).

### 3.2 Disproportionality Analysis Identifies Critical PN‐Related AEs Associated With FQ Treatment

**FIGURE 2:**
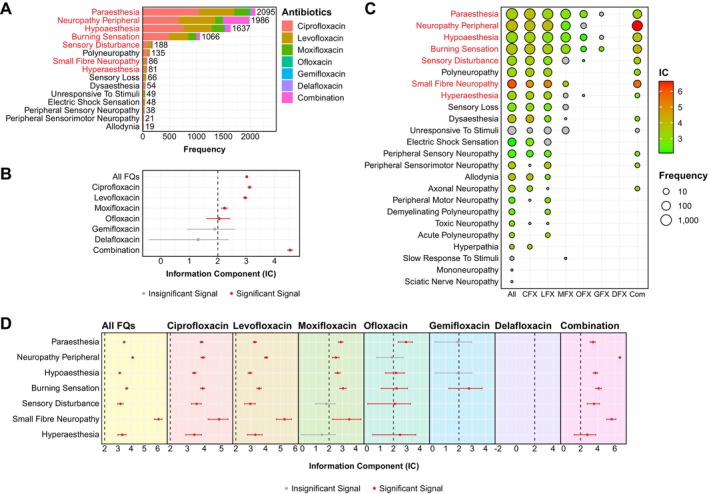
Quantitative evaluation of FQ‐associated PN‐related signals via disproportionality analysis using real‐world from FAERS database. (A) Bar chart illustrating the 15 most common PN‐related AEs, color‐coded by individual FQ. (B) Disproportionality analysis using information component (IC) values identified significant PN‐related signals for ciprofloxacin, levofloxacin, moxifloxacin, ofloxacin, and combination therapy. (C) Heat map depicting IC values across all PN‐related AEs. Dots represent PN‐related preferred terms (PTs) with at least three reported cases in FAERS, with dot size proportional to frequency for individual agents. PN‐related PT terms highlighted in red indicate common positive disproportionality signals shared by at least three FQs (CFX, ciprofloxacin; Com, combination therapy; DFX, delafloxacin; GFX, gemifloxacin; LFX, levofloxacin; MFX, moxifloxacin; OFX, ofloxacin). (D) Forest plots of IC values (with 95% confidence intervals; CI) of common PN‐related signals shared by at least three FQs. Red denoted significant positive signals whereas gray indicated insignificant signals.

The most frequent FQ‐associated PN‐related manifestations included paraesthesia [2095/5588 (37.49%)], followed by peripheral neuropathy [1986/5588 (35.54%)], hypoaesthesia [1637/5588 (29.29%)] and burning sensation [1066/5588 (19.08%)] (Figure 2A; Table S7). To systematically detect PN‐related signals across different FQs, we conducted disproportionality analyses for each FQ by calculating the information component (IC) using a Bayesian confidence propagation neural network (BCPNN) signal detection method, with the complete FAERS database (2007–2024 Q3) as a comparator (Table 1) [9]. Significant signals were defined as those with an IC exceeding 2 and lower limits of 95% CI greater than 0 (see Section 2), indicating disproportionate reporting that warranted further investigation. FQ use demonstrated disproportionate reporting of PN, evidenced by a high overall IC value [3.02 (2.98–3.05); *p <* 0.0001]. Among all six FDA‐approved FQs, positive disproportionality signals were detected for ciprofloxacin [3.12 (3.06–3.17); *p <* 0.0001], levofloxacin [2.97 (2.91–3.02); *p <* 0.0001], moxifloxacin [2.24 (2.13–2.34); *p <* 0.0001], and ofloxacin [2.05 (1.62–2.42); *p =* 4.60 × 10^−16^] (Figure 2B). Concurrent use of two or more FQs (i.e., combination therapy) generated the strongest IC signal [4.54 (4.46–4.62); *p <* 0.0001] (Figure 2B).

**TABLE 1:** Disproportionality analysis of PN‐related adverse event (AE) safety signals for six FDA‐approved fluoroquinolones (FQs).

Antibiotics	Case counts with PN‐related AEs	IC (95% CI)	*p*
All FQs	5588/37,778	3.02	*p* < 0.0001
		(2.98–3.05)	
Ciprofloxacin	2291/14,434	3.12	*p* < 0.0001
		(3.06–3.17)	
Levofloxacin	1942/13,598	2.97	*p* < 0.0001
		(2.91–3.02)	
Moxifloxacin	642/7439	2.24	*p* < 0.0001
		(2.13–2.34)	
Ofloxacin	46/588	2.05	4.60E−16
		(1.62–2.42)	
Gemifloxacin	11/142	1.89	6.71E−05
		(0.95–2.59)	
Delafloxacin	4/72	1.31	0.04
		(−0.39–2.36)	
Combination	652/1505	4.54	*p* < 0.0001
		(4.46–4.62)	

*Note:* The table presents case counts for individual FQs and their information component (IC) values with 95% confidence intervals (CI).

To further probe for any previously unrecognized PN‐related AEs associated with FQ treatment, we performed disproportionality analysis on each PN‐related PT with “Nervous System Disorders” as primary system organ class (SOC) and three or more cases (*N* = 24/28 PTs). Of these, 21 PN‐related PTs showed positive signals following FQ use in the overall context (Figure 2C; see Table S8 for the complete disproportionality analysis). Among all six FDA‐approved FQs, levofloxacin (17/24) and ciprofloxacin (16/24) demonstrated the most potential signals with IC values exceeding 2, followed by moxifloxacin (6/24), ofloxacin (5/24) and gemifloxacin (1/24). Notably, whilst gemifloxacin received considerably less attention regarding its potential neurotoxicity compared with other well‐established FQs, our disproportionality analysis uncovered a positive signal for burning sensation [2.76 (1.24–3.74); *p =* 1.86 × 10^−5^], a hallmark PN symptom not commonly associated with gemifloxacin [11, 12]. Despite being based on limited reports, this signal warrants further validation in large, independent datasets. Combination FQ therapy yielded 12 positive signals (out of 24), with peripheral neuropathy showing the strongest IC signal [6.61 (6.50–6.71); *p <* 0.0001] (Figure 2C).

To identify the most representative FQ‐associated PN signals, we selected PTs with strong IC signals (> 2) in the overall context and positive signals in at least three individual FQs. Finally, seven PTs (paraesthesia, peripheral neuropathy, hypoaesthesia, burning sensation, sensory disturbance, small fiber neuropathy, and hyperaesthesia) demonstrated disproportionate reporting patterns suggestive of association with FQ treatment and were consequently prioritized as FQ‐associated PN‐related signals warranting further clinical and epidemiological investigation (Figure 2D).

### 3.3 Characterization of FQ‐Associated PN‐Related Adverse Events

**FIGURE 3:**
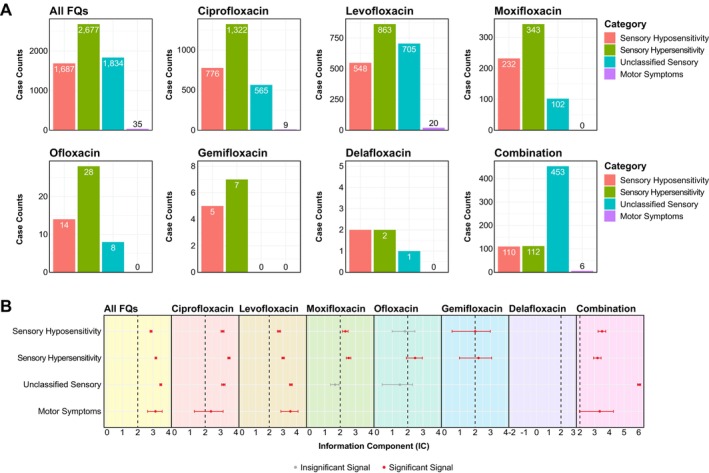
Disproportionality analysis of distinct PN categories associated with FQ treatment. (A) PN‐related preferred terms (PTs) were categorized into “Sensory Hyposensitivity,” “Sensory Hypersensitivity,” “Unclassified Sensory” and “Motor Symptoms.” Bar plots depict the frequency of each category across individual FQs. (B) Disproportionality analysis conducted on each category for six FDA‐approved FQs using information components (ICs). Forest plots showed IC values (with 95% confidence intervals; CI) of disproportionality signals for each category across individual FQs. Red denoted significant positive signals whereas gray indicated insignificant signals.

To further delineate the sensory and motor manifestations associated with FQ use, we categorized PN‐related PTs into four groups: sensory hyposensitivity (e.g., paraesthesia, hypoaesthesia, sensory loss or unresponsive to stimuli), sensory hypersensitivity (e.g., burning sensation, hyperaesthesia, electric shock sensation or allodynia) [13], unclassified sensory symptoms, and motor symptoms (Table S9). As FAERS database lacks detailed clinical indications or symptom descriptions for individual cases, our classification relied solely on PTs documented in each AER. Consequently, PTs such as sensory disturbance, axonal neuropathy and small fiber neuropathy, which may present with sensory loss, pain hypersensitivity or both, were designated as “unclassified sensory symptoms.” Similarly, only PTs with explicit motor involvement (e.g., peripheral motor neuropathy and peripheral sensorimotor neuropathy) were classified as “motor symptoms,” whilst excluding PTs such as axonal neuropathy from this category where motor involvement remains uncertain. Among all FQ‐associated AERs with PN‐related AEs, 2677 cases (47.91%) exhibited pain/sensory hypersensitivity symptoms, whilst 1687 cases (30.19%) presented with sensory hyposensitivity and sensory loss. Motor manifestations were explicitly documented in only 35 cases (0.63%), specifically restricted to those diagnosed with motor neuropathy (Figure 3A). It is important to note that these figures likely underestimated the actual occurrence of motor symptoms, as patients diagnosed with PN or other forms of neuropathy may also experience motor symptoms that were not captured by some generalized PTs in the FAERS database.

To quantitatively assess potential disproportionality patterns between FQ use and PN‐related symptom categories, we calculated ICs for each category across individual FQs (Table 2). Disproportionality analysis revealed positive pharmacovigilance signals for all three sensory symptom categories, with the highest IC values observed for unclassified sensory symptoms [IC: 3.50 (3.44–3.56); *p <* 0.0001], followed by sensory hypersensitivity [3.18 (3.13–3.23); *p <* 0.0001] and sensory hyposensitivity [2.87 (2.80–2.93); *p <* 0.0001] (Figure 3B). Despite the small number of reports, disproportionate reporting [3.16 (2.64–3.59); *p* = 2.47 × 10^−23^] was still detected for motor symptoms associated with FQ use. Analysis of individual FQs revealed that ciprofloxacin, levofloxacin and combination therapy generated positive disproportionality signals across all four symptom categories (Table 2). Moxifloxacin, ofloxacin and gemifloxacin demonstrated positive signals for sensory hypersensitivity and/or hyposensitivity, with no disproportionate reporting of motor symptoms detected in the analysis (Figure 3B). No signal was detected for delafloxacin, likely due to the low prescribing rate and small number of AERs within the dataset.

**TABLE 2:** Disproportionality analysis of PN‐related AE categories for six FDA‐approved fluoroquinolones (FQs).

Category	Case Counts	IC (95% CI)	*p*	Case Counts	IC (95% CI)	*p*	Case Counts	IC (95% CI)	*p*	Case Counts	IC (95% CI)	*p*
	**Total (*N* = 37,778)**			**Ciprofloxacin (*N* = 14,434)**			**Levofloxacin (*N* = 13,598)**			**Moxifloxacin (*N* = 7439)**		
Sensory Hyposensitivity	1687	2.87	*p* < 0.0001	776	3.13	*p* < 0.0001	548	2.71	4.63E−253	232	2.34	2.08E−86
		(2.80–2.93)			(3.03–3.22)			(2.59–2.83)			(2.15–2.51)	
Sensory Hypersensitivity	2677	3.18	*p* < 0.0001	1322	3.55	*p* < 0.0001	863	3.02	*p* < 0.0001	343	2.55	1.39E−145
		(3.13–3.23)			(3.47–3.62)			(2.92–3.11)			(2.40–2.70)	
Unclassified Sensory	1834	3.50	*p* < 0.0001	565	3.18	*p* < 0.0001	705	3.59	*p* < 0.0001	102	1.66	3.23E−23
		(3.44–3.56)			(3.06–3.30)			(3.48–3.69)			(1.37–1.93)	
Motor Symptoms	35	3.16	2.47E−23	9	2.37	1.09E−05	20	3.55	1.08E−17	0	N/A	N/A
		(2.64–3.59)			(1.29–3.17)			(2.85–4.11)				
	**Ofloxacin (*N* = 588)**			**Gemifloxacin (*N* = 142)**			**Delafloxacin (*N* = 72)**			**Combination (*N* = 1505)**		
Sensory Hyposensitivity	14	1.82	2.30E−05	5	2.01	1.92E−03	2	1.41	7.23E−02	110	3.51	2.48E−78
		(0.99–2.47)			(0.50–2.99)			(−1.17–2.75)			(3.23–3.76)	
Sensory Hypersensitivity	28	2.48	8.61E−14	7	2.22	1.41E−04	2	1.23	1.09E−01	112	3.20	1.06E−69
		(1.92–2.96)			(0.98–3.07)			(−1.34–2.57)			(2.93–3.44)	
Unclassified Sensory	8	1.49	4.33E−03	0	N/A	N/A	1	0.89	2.66E−01	453	6.03	*p* < 0.0001
		(0.33–2.32)						(−2.93–2.52)			(5.91–6.14)	
Motor Symptoms	0	N/A	N/A	0	N/A	N/A	0	N/A	N/A	6	3.35	8.74E−09
											(1.97–4.28)	

*Note:* The table reports case counts per category for individual FQs, and corresponding information component (IC) values with 95% confidence intervals (CI).

### 3.4 Time‐To‐Onset (TTO) Analysis of FQ‐Associated PN‐Related AERs

**FIGURE 4:**
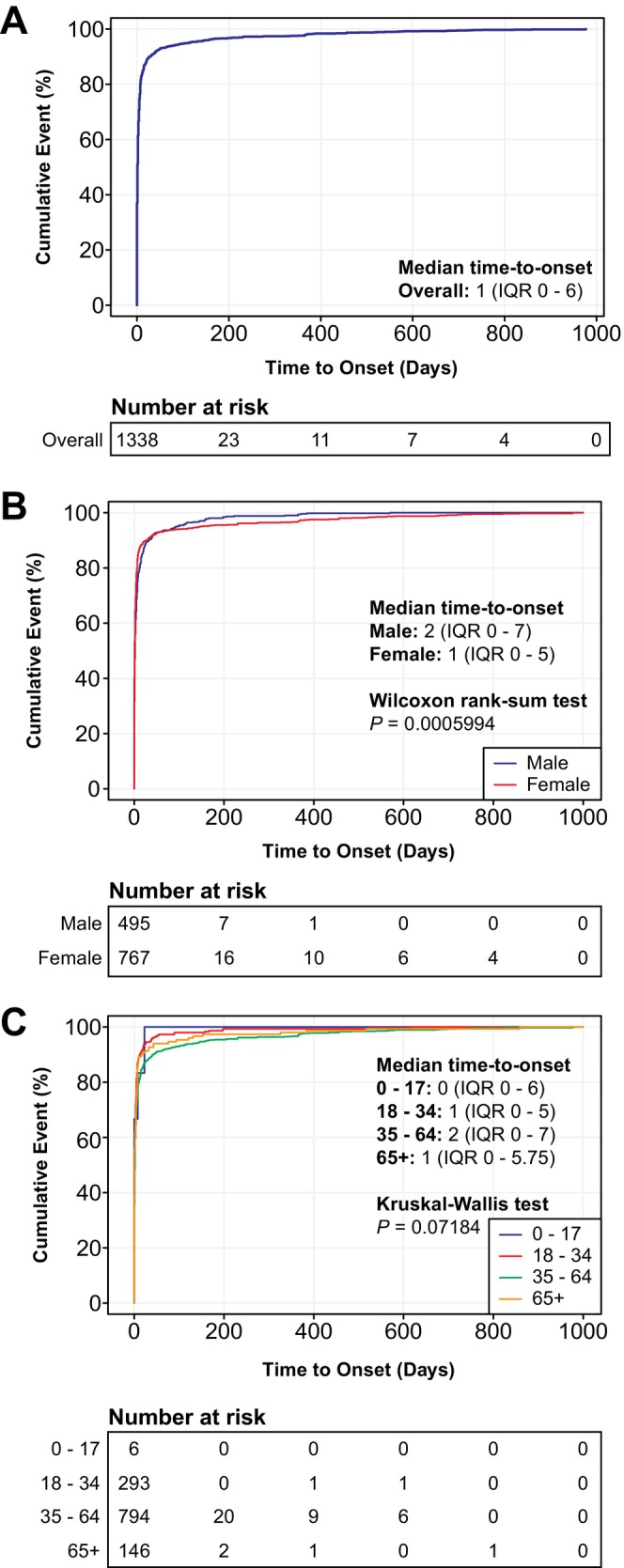
Time‐to‐onset (TTO) analysis on PN‐related AEs associated with FQ treatment. (A) Cumulative distribution curve showing the overall onset time of PN‐related AEs following FQ treatment based on FAERS database. (B) Cumulative distribution curves demonstrating the onset time of PN‐related AEs across both sexes. (C) Cumulative distribution curves illustrating the onset time of PN‐related AEs across different age groups. Statistical analyses were performed using the Wilcoxon rank sum test in (B) and the non‐parametric Kruskal–Wallis test in (C).

Among all 5588 FQ‐associated AERs with PN‐related AEs, TTO analysis was feasible for 1383 reports (24.7%) documented both the medication start dates and dates of first AE onset. The majority of FQ‐associated PN‐related AEs [1109/1383 (80.2%)] occurred within 7 days of treatment initiation [median: 1 day (IQR: 0–6)] (Figure 4A). Females exhibited a modestly but significantly shorter onset time [median: 1 (0–5)] compared with males [median: 2 (0–7)] (Figure 4B). Notably, children and adolescents (0–17 years) demonstrated the shortest onset time [median: 0 (0–6)], middle‐aged (35–64 years) individuals experienced slightly longer onset time [median: 2 (0–7)] than young (18–34 years) [median: 1 (0–5)] and elderly (≥ 65 years) [median: 1 (0–5.75)] populations (Figure 4C). It is important to note that the FAERS database did not systematically capture outcome information regarding whether PN‐related AEs were resolved, improved, or persisted following onset.

### 3.5 Factors Influencing FQ‐Associated PN Adverse Events

**FIGURE 5:**
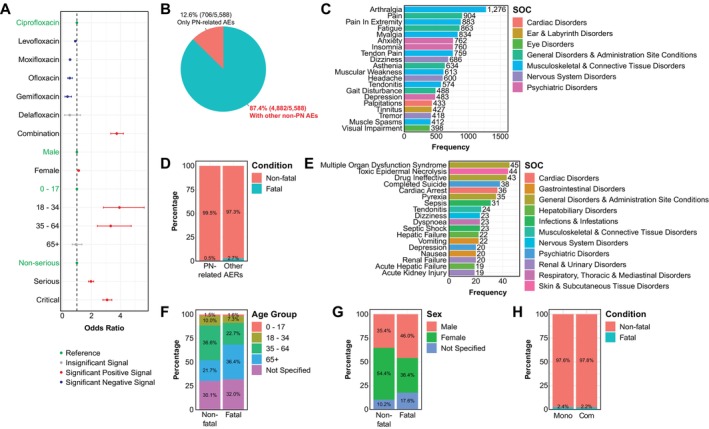
Factors influencing the occurrence and outcomes of FQ‐associated AERs. (A) Forest plot summarizing univariate logistic regression analyses of risk factors that influenced the occurrence of PN‐related AEs associated with FQ treatment. Red denoted significant positive signals, blue indicated significant negative signals, gray represented insignificant signals, and green indicated reference factors. (B) The majority of FQ‐associated AERs with PN‐related AEs were accompanied by concurrent non‐PN AEs. (C) Bar plot showing the most frequent non‐PN AEs concurrent in FQ‐associated AERs with PN‐related AEs, with colors indicating their primary System Organ Class (SOC). (D) FQ‐associated AERs with PN‐related AEs displayed a relatively lower mortality compared with those exhibiting other non‐PN AEs. (E) Bar plot illustrating the most common PTs observed in fatal FQ‐associated AERs with PN‐related AEs, with colors indicating their primary System Organ Class (SOC). (F, G) Elderly individuals (≥ 65 years) (F) and males (G) constituted the majority of fatal FQ‐associated AERs with PN‐related AEs. (H) Mortality risks were comparable between patients receiving either FQ monotherapy or combination therapy comprising two or more FQs.

To further explore reporting patterns of FQ‐associated PN‐related AEs, we conducted univariate logistic regression analyses, calculating odds ratios (ORs) based on all FQ‐associated AERs (Table S10) [9]. Using ciprofloxacin as the reference, four FDA‐approved FQs demonstrated potentially lower ORs: levofloxacin [OR: 0.89 (0.84–0.96); *p* = 0.017], moxifloxacin [0.55 (0.49–0.61); *p* = 4.23 × 10^−28^], ofloxacin [0.51 (0.37–0.69); *p* = 2.78 × 10^−4^], and gemifloxacin [0.36 (0.18–0.63); *p* = 0.014], whereas delafloxacin showed no substantial difference [0.51 (0.15–1.24); *p* > 0.05]. Notably, combination therapy with two or more FQs demonstrated higher odds of reporting PN‐related AEs compared with ciprofloxacin monotherapy [3.74 (3.33–4.20); *p* = 1.52 × 10^−107^] (Figure 5A). Sex appeared to influence reporting patterns of FQ‐associated PN‐related AEs, with females more frequently represented than males [1.12 (1.05–1.19); *p* = 0.006] (Figure 5A). Compared with children and adolescents (0–17 years), young (18–34 years) and middle‐aged (35–64 years) individuals were more frequently represented in PN‐related AERs, with ORs of 3.93 (2.83–5.63; *p* = 7.85 × 10^−14^) and 3.32 (2.41–4.74; *p* = 4.00 × 10^−11^), respectively, whereas the elderly population (≥ 65 years) showed no substantial difference [0.94 (0.67–1.35); *p* > 0.05] to the reference group (0–17 years) (Figure 5A). Additionally, patients experiencing severe adverse reactions with serious [1.97 (1.80–2.16); *p* = 1.18 × 10^−46^] or critical (life‐threatening or death) outcomes [3.08 (2.79–3.40); *p* = 7.08 × 10^−107^] were more frequently reported among PN‐related AERs compared with non‐serious cases (Figure 5A).

Among 5588 AERs with PN‐related AEs, 4882 reports (87.37%) presented with concurrent AEs, whilst 706 cases (12.63%) manifested PN‐related AEs alone (Figure 5B). Analysis of the 20 most frequent concurrent AEs (Figure 5C) revealed musculoskeletal and connective tissue disorders (*N* = 5351) as the predominant SOC category, followed by general disorders and administration site conditions (*N* = 2889) and psychiatric disorders (*N* = 2005). We also detected other neurological manifestations (*N* = 1704) that frequently co‐occur with PN‐related AEs, including dizziness (*N* = 686), headache (*N* = 600), and tremor (*N* = 418) (Figure 5C).

### 3.6 Reporting Patterns of Fatal Outcomes in FQ‐Associated Adverse Event Reports

During 2007–2024 Q3, 898 reports with fatal outcomes were documented among all FQ‐associated AERs (Table S11). Among these, 25 also included PN‐related AEs (Figure 5D). The most frequent AEs in fatal cases were multiple organ dysfunction syndrome (*N* = 45), followed by toxic epidermal necrolysis (*N* = 44), and drug ineffectiveness (*N* = 43). No PN‐related AEs were featured among the most common AEs in cases with mortality (Figure 5E). Notably, suicidal behaviors were represented in 38 fatal cases (4.23%), whilst 20 cases (2.23%) exhibited signs of depression (Figure 5E).

Further analyses suggested differences in reporting patterns of fatal outcomes in FQ‐associated AERs across age and sex (Table S12). The elderly (≥ 65 years) accounted for a higher proportion of fatal reports [327/898 (36.41%)] compared with other age groups including children and adolescents (0–17 years) [14/898 (1.56%)], young adults (18–34 years) [66/898 (7.35%)], and middle‐aged adults (35–64 years) [204/898 (22.72%)] (*p* = 3.47 × 10^−29^) (Figure 5F). Although univariate logistic regression analysis indicated increased odds of PN‐related AEs in females following FQ use (Figure 5A), males represented a higher proportion of reports with fatal outcomes [413/898 (45.99%)] than females [327/898 (36.41%)] (*p* = 2.52 × 10^−28^) (Figure 5G). Interestingly, combination therapy with two or more FQs did not appear to increase reporting of fatal outcomes, as the proportion of fatal cases in combination therapy [33/1505 (2.19%)] was comparable to monotherapy [865/36,273 (2.38%)] (*p* = 0.69) (Figure 5H).

## 4 Discussion

FQ antibiotics, among the most widely prescribed worldwide and essential for treating various bacterial infections [3], have raised substantial regulatory concerns due to their links to severe and irreversible PN [4]. Here, we presented a comprehensive global pharmacovigilance analysis of FQ‐associated PN‐related AEs using real‐world data from the FAERS database (2007–2024 Q3). Our disproportionality analysis identified seven PN‐related AEs frequently reported following FQ use, highlighting potential signals involving both sensory and motor manifestations. Among all FDA‐approved FQs, ciprofloxacin exhibited the highest odds of being reported in PN‐related AEs, while combination therapy involving multiple FQs was associated with disproportionately higher reporting frequencies. Although females and adults aged 18–64 years were more frequently represented in PN‐related AEs, males and elderly patients (≥ 65 years) accounted for a greater proportion of FQ‐associated AERs with fatal outcomes.

Our temporal analysis identified patterns suggestive of stimulated reporting following the introduction of the boxed warning in 2016 [14]. During 2016–2019, cases of FQ‐associated AERs showed a gradual increase, followed by a subsequent decline. This pattern might reflect heightened awareness prompted by this regulatory action [6]. It appeared most pronounced in 2016, when 27.0% of FQ‐associated cases displayed PN‐related AEs, with both the absolute number and proportion declining thereafter. Whilst our analysis demonstrated a downward trend post‐2016, continued surveillance of FQ‐associated reporting patterns remains important to understand whether prescription restrictions implemented by regulatory authorities have translated into reduced prescribing practices. Alarmingly, developing regions in the Middle East and South Asia have recorded substantial increases in FQ prescriptions [3], with misuse remaining prevalent in countries such as India and China [15, 16]. Given the therapeutic efficacy of FQ, complete prohibition remains impractical without comparable alternatives. When FQ treatment becomes unavoidable, particularly for severe bacterial infections, rigorous patient safety monitoring programmes are essential due to the increased reporting of PN‐related AEs [17]. However, objective assessment of PN symptoms presents challenges common to various drug‐induced PN, including chemotherapy‐induced peripheral neuropathy (CIPN) [18] and FQ‐induced PN. For non‐hospitalized patients receiving FQs from community prescribers, remote monitoring via mobile applications may offer viable solutions for active PN surveillance [19].

The historically perceived “safety” of FQ treatment in elderly patients requires substantial revision. Elderly patients exhibited higher plasma concentrations of renally excreted FQs due to age‐associated renal decline [20], often necessitating dose reductions to prevent drug accumulation. Our pharmacovigilance analysis, whilst not identifying advanced age as a prominent feature in reports of FQ‐associated PN‐related AEs, likely reflects substantial under‐reporting. PN symptoms following FQ treatment may be erroneously attributed to age‐associated decline, particularly given the high prevalence of concurrent conditions (e.g., increased body mass index, diabetes, vitamin B12 deficiency and rheumatoid arthritis) linked with sensory deficits in this population [21]. Additionally, our analysis showed that fatal outcomes were more commonly reported in patients with advancing age following FQ treatment, consistent with a recent study [22]. Although our study could not provide absolute quantification of risks, these reporting trends underscore the need for cautious prescribing in elderly populations, particularly in long‐term care facilities where FQs remain one of the most commonly used antibiotic classes [23]. Such observations reinforce the importance of strengthened antimicrobial stewardship programmes in these settings [24].

The current study suggested modest sex‐based differences in reporting of FQ‐associated PN‐related AEs, with a higher proportion of FQ‐associated reports involving females. Whilst this observation aligns with prior evidence linking female sex to increased reporting of painful diabetic PN [25], alcohol‐induced PN [26], and CIPN [27] triggered by neurotoxic chemotherapeutic agents [28, 29, 30], interpretation requires caution given potential reporting bias and lack of detailed clinical information in FAERS. Although previous research indicated that females may be more susceptible to certain FQ‐associated AEs such as aortic aneurysm and dissection [31], current findings represent hypothesis‐generating signals and are not indicative of confirmed associations. Notably, despite being less frequently represented in PN‐related AERs, males more commonly appeared in reports with fatal outcomes following FQ exposure. Whether this pattern reflects true sex‐related differences or reporting artifacts remains uncertain and merits follow‐up studies.

Concurrent administration of multiple FQs is generally discouraged owing to overlapping mechanisms of action and potential cumulative toxicity [32]. Although simultaneous use of more than one FQ is not supported by strong clinical evidence, such prescribing may occur in attempts to broaden antimicrobial coverage in complex or polymicrobial infections [33]. In our FAERS dataset, 1505 cases involved combination FQ therapy, of which 652 (43.32%) reported PN‐related AEs. However, the rationale for concurrent prescribing was not captured in FAERS. The apparently elevated proportion of PN‐related AERs in this subgroup requires careful interpretation, as unusual prescribing patterns typically generate disproportionate reporting in spontaneous reporting systems including FAERS [6]. Patients receiving multiple antimicrobials may have more severe infections or comorbidities that independently influence AE reporting [6]. Whilst our analysis showed similar reporting frequencies of fatal outcomes between combination therapy with two or more FQs and FQ monotherapy, the higher reporting of PN‐related AEs in this subgroup merits further evaluation through controlled pharmacoepidemiological studies that could account for disease severity, comorbidity burden, and prescribing context. Treatment decisions for complicated infections should continue to prioritize established evidence‐based regimens (e.g., ceftolozane with tazobactam and/or metronidazole), where appropriate [34].

The mechanisms underpinning FQ‐associated PN remained largely uncharacterised [4]. Research highlighted FQ‐associated molecular changes in mitochondrial function, collagen production, and GABA antagonism [35, 36, 37]. Interestingly, we and other demonstrated that chemotherapeutic agents induced off‐targeted effects to cause mitochondrial dysfunction (e.g., disrupted axonal mitochondrial transport and mitochondrial swelling) in peripheral neurons, a mechanism linked to CIPN pathogenesis [28, 29, 30] and resembling cellular changes observed after FQ treatment. Elucidating the molecular basis of FQ‐induced pathological changes in sensory neurons, alongside developing appropriate in vitro (e.g., primary sensory neuronal cultures [38, 39, 40] and patients‐derived induced pluripotent stem cells [41]) and in vivo disease models [28], could provide novel mechanistic insights. Additionally, testing readily available compounds (e.g., mitochondria‐targeted M1 compound) that restore mitochondrial dynamics in peripheral neurons [42, 43] may offer therapeutic strategies to mitigate the risk of FQ‐induced PN and enable safer clinical use.

This study has several important limitations inherent to spontaneous reporting systems. FAERS database relies solely on voluntary reporting, which introduces reporting bias and precludes true incidence estimations. The observed disproportionality signals likely reflected reporting patterns rather than actual PN occurrence rates. Recent regulatory warnings (e.g., 2016 FDA boxed warning) regarding FQ‐associated PN might have heightened clinical awareness, potentially amplifying signals through stimulated reporting bias [6, 14]. Critical confounding factors, including dosage regimens, medical history, and comorbidities, were inconsistently documented in the dataset [44, 45]. Notably, various established PN risk factors, including diabetes, HIV infection, alcohol consumption, chemotherapy exposure, and nutritional deficiencies, were not systematically captured [25, 26, 28, 46, 47]. Consequently, our analyses were constrained by the inability to fully account for these critical confounders within multivariate logistic regression models. We therefore used univariate logistic regression only as an exploratory tool to characterize reporting patterns by individual FQs and demographic subgroups [10]. Future analyses could employ stratified approaches using available variables (e.g., age, sex and reporting country) to provide additional insights, though limited by unmeasured confounding. The observed “burning sensation” signal for gemifloxacin was based on limited AERs, requiring cautious interpretation and further validation in large, independent pharmacovigilance datasets from the EU and UK where the agent remains available. Our decision to restrict analyses on reports involving FQ treatment only aimed to minimize confounding but might have introduced selection bias by excluding patient populations with higher polypharmacy rates, particularly elderly patients with multiple comorbidities who may represent a substantial proportion of FQ users. This limits generalizability to real‐world clinical populations where polypharmacy is common. The shorter post‐marketing history of delafloxacin (approved in 2017) also limited the completeness of its reporting profile. Despite these limitations, the observed reporting trends offered preliminary, hypothesis‐generating signals that justified further evaluation through prospective cohort studies or comprehensive electronic health record databases capable of supporting more robust confounder adjustment.

In conclusion, our FAERS‐based pharmacovigilance analysis identified disproportionality signals for PN‐related AERs associated with FDA‐approved FQs. Despite regulatory restrictions implemented in 2016, reporting rates remained substantial over the 17‐year study period, though a downward trend emerged in recent years. These findings represented hypothesis‐generating signals that required validation through controlled epidemiological studies with adequate confounder adjustment. Healthcare providers should remain vigilant for PN symptoms in patients receiving FQ therapy. Understanding the molecular mechanisms of FQ‐induced PN could guide the development of targeted neuroprotective strategies and enable repurposing of existing drugs for risk mitigation through transcriptomic signature matching using Connectivity Map [48] or Library of Integrated Network‐Based Cellular Signatures (LINCS) databases [49, 50].

## Author Contributions

N.P.B.A., R.B., and J.B. wrote the manuscript. N.P.B.A. and R.B. designed the research. N.S., K.D., H.‐T.C., R.B. and N.P.B.A. performed the research and analyzed the data.

## Funding

This research was supported by Rosetrees/Stoneygate Trust (ref. no.: Seedcorn2024\100044) and Royal Society Research Grant (ref. no.: RG\R1\251126) awarded to N.P.B.A., and Royal Society Research Grant (ref. no.: RGS\R2\192126) awarded to R.A.B.

## Conflicts of Interest

N.P.B.A. served as a member of the Faculty Ethics Committee at the Faculty of Science and Health during his tenure at the University of Portsmouth; however, he had no influence on the ethical approval of this study. All other authors declared no competing interests for this work.

## Supporting information


**Figure S1:** Flow chart illustrating the selection process of fluoroquinolone (FQ)‐associated adverse event reports (AERs). The complete dataset, comprising AERs in which any of six FDA‐approved FQs (ciprofloxacin, levofloxacin, moxifloxacin, ofloxacin, gemifloxacin, and delafloxacin) were listed as suspect drugs, was extracted from the FDA Adverse Event Reporting System (FAERS) Public Dashboard (2007–2024 Q3). Following deduplication and exclusion of AERs involving concomitant medications, a total of 37,778 cases were retained for descriptive and disproportionality analyses. Among these, 5588 cases presented with peripheral neuropathy (PN)‐related adverse events (AEs).
**Figure S2:** Demographic characteristics of fluoroquinolone (FQ)‐associated adverse event reports (AERs) from the FAERS database (2007–2024 Q3). Proportional bar plots depicting distributions of (A) age group, (B) sex, (C) reporter type, (D) country of origin, (E) seriousness classification, and (F) clinical outcomes across all six FDA‐approved fluoroquinolones (CFX, ciprofloxacin; Com, combination therapy; DFX, delafloxacin; GFX, gemifloxacin; LFX, levofloxacin; MFX, moxifloxacin; OFX, ofloxacin).
**Figure S3:** Demographic characteristics of FQ‐associated AERs with peripheral neuropathy (PN)‐related adverse events (AEs) from the FAERS database (2007–2024 Q3). Proportional bar plots demonstrating distributions of (A) age group, (B) sex, (C) reporter type, (D)country of origin, (E) seriousness classification, and (F) clinical outcomes for all six FDA approved FQs with PN‐related AEs (CFX, ciprofloxacin; Com, combination therapy; DFX, delafloxacin; GFX, gemifloxacin; LFX, levofloxacin; MFX, moxifloxacin; OFX, ofloxacin). (G) Clinical indications for FQ usage in these FQ‐associated AERs with PN‐related AEs are presented in descending order of frequency, with bacterial infections appeared as the majority of prescribing patterns.
**Table S1:** Fluoroquinolones (FQs) investigated in the current study. This table presents the approval status of each FQ by the FDA, EMA and MHRA, alongside the nomenclature used for enquiries from the FAERS Public Dashboard.
**Table S2:** Total prescriptions of the most commonly used FQs in the United States from 2013 to 2022. Of the six FDA‐approved FQs, four (ciprofloxacin, levofloxacin, moxifloxacin and ofloxacin) featured in the Top 300 Drug List, representing the most commonly prescribed FQs in the US. Data were extracted from the ClinCalc DrugStats Database (version 2024.08). All medication utilization data originated from the annual Medical Expenditure Panel Survey (MEPS), conducted by the Agency for Healthcare Research and Quality (AHRQ) under the US government.
**Table S3:** Neurological characteristics of FQ‐associated adverse event reports (AERs).

**Table S4:** Comprehensive list of clinical indications for FQ use in FQ associated AERs with peripheral neuropathy (PN)‐related adverse events (AEs).
**Table S5:** Demographic characteristics of FQ‐associated AERs. Data derived from FAERS database, 2007–2024 Q3.
**Table S6:** Demographic characteristics of FQ‐associated AERs with PN‐related AEs. Data derived from FAERS, 2007–2024 Q3.
**Table S7:** Case counts for each PN‐related preferred term (PT) by individual FQs.

**Table S8:** Disproportionality analysis of PN‐related PTs by individual FQs.
**Table S9:** Classification for PN‐related preferred terms (PTs).
**Table S10:** Univariate Logistic regression analysis of the odds ratio for FQ‐associated PN‐related AEs.
**Table S11:** Case counts of FQ‐associated AERs with fatal outcomes.
**Table S12:** Clinical characteristics of FQ‐associated PN‐related AERs with fatal outcomes.

**Data S1:** The READUS‐PV checklist.

**Data S2:** The READUS‐PV checklist for abstracts.

## Data Availability

All data generated or analyzed in this study are included in the manuscript and its Supporting Information. All AERs are publicly available and can be retrieved directly from the FAERS Public Dashboard (https://www.fda.gov/drugs/fdas‐adverse‐event‐reporting‐system‐faers/fda‐adverse‐event‐reporting‐system‐faers‐public‐dashboard).

## References

[1] I.‐A. Lungu , O.‐L. Moldovan , V. Biriș , and A. Rusu , “Fluoroquinolones Hybrid Molecules as Promising Antibacterial Agents in the Fight Against Antibacterial Resistance,” Pharmaceutics 14 (2022): 1749. 36015376 10.3390/pharmaceutics14081749 PMC9414178

[2] S. P. Umarje , C. G. Alexander , and A. J. Cohen , “Ambulatory Fluoroquinolone Use in the United States, 2015‐2019,” Open Forum Infectious Diseases 8 (2021): ofab538, 10.1093/ofid/ofab538. 34901300 PMC8659351

[3] A. J. Browne , M. G. Chipeta , G. Haines‐Woodhouse , et al., “Global Antibiotic Consumption and Usage in Humans, 2000–18: A Spatial Modelling Study,” Lancet Planetary Health 5 (2021): e893–e904. 34774223 10.1016/S2542-5196(21)00280-1 PMC8654683

[4] C. Bove , R. A. Baldock , O. Champigneulle , L. Martin , and C. L. Bennett , “Fluoroquinolones: Old Drugs, Putative New Toxicities,” Expert Opinion on Drug Safety 21 (2022): 1365–1378. 36384376 10.1080/14740338.2022.2147924

[5] D. Morales , A. Pacurariu , J. Slattery , L. Pinheiro , P. McGettigan , and X. Kurz , “Association Between Peripheral Neuropathy and Exposure to Oral Fluoroquinolone or Amoxicillin‐Clavulanate Therapy,” JAMA Neurology 76 (2019): 827–833. 31034074 10.1001/jamaneurol.2019.0887 PMC6583699

[6] E. Potter , M. Reyes , J. Naples , and G. Dal Pan , “FDA Adverse Event Reporting System (FAERS) Essentials: A Guide to Understanding, Applying, and Interpreting Adverse Event Data Reported to FAERS,” Clinical Pharmacology and Therapeutics 118 (2025): 567–582, 10.1002/cpt.3701. 40384638 PMC12393772

[7] M. Fusaroli , F. Salvo , B. Begaud , et al., “The REporting of A Disproportionality Analysis for DrUg Safety Signal Detection Using Individual Case Safety Reports in PharmacoVigilance (READUS‐PV): Explanation and Elaboration,” Drug Safety 47 (2024): 585–599, 10.1007/s40264-024-01423-7. 38713347 PMC11116264

[8] P. M. Tregunno , D. B. Fink , C. Fernandez‐Fernandez , E. Lazaro‐Bengoa , and G. N. Noren , “Performance of Probabilistic Method to Detect Duplicate Individual Case Safety Reports,” Drug Safety 37 (2014): 249–258, 10.1007/s40264-014-0146-y. 24627310

[9] Y. Chen , X. Ren , Y. Dai , and Y. Wang , “Pharmacovigilance Study of the Association Between Peripheral Neuropathy and Antibody‐Drug Conjugates Using the FDA Adverse Event Reporting System,” Scientific Reports 14 (2024): 21386, 10.1038/s41598-024-71977-0. 39271716 PMC11399297

[10] H.‐T. Chau and N. P. B. Au , “Fluoroquinolone‐Associated Psychiatric and Ocular Adverse Events: A Disproportionality Analysis Using Real‐World Data From FAERS (2011–2024),” Pharmacology Research & Perspectives 14 (2026): e70206, 10.1002/prp2.70206. 41410077 PMC12712350

[11] M. J. Barrett and I. S. Login , “Gemifloxacin‐Associated Neurotoxicity Presenting as Encephalopathy,” Annals of Pharmacotherapy 43 (2009): 782–784. 19276313 10.1345/aph.1L621

[12] M. F. Grill and R. K. Maganti , “Neurotoxic Effects Associated With Antibiotic Use: Management Considerations,” British Journal of Clinical Pharmacology 72 (2011): 381–393. 21501212 10.1111/j.1365-2125.2011.03991.x PMC3175508

[13] R. Baron , C. Maier , N. Attal , et al., “Peripheral Neuropathic Pain: A Mechanism‐Related Organizing Principle Based on Sensory Profiles,” Pain 158 (2017): 261–272, 10.1097/j.pain.0000000000000753. 27893485 PMC5266425

[14] G. J. Dal Pan , M. Lindquist , and K. Gelperin , “Postmarketing Spontaneous Pharmacovigilance Reporting Systems,” Pharmacoepidemiology 25 (2019): 165–201.

[15] G. Iacobucci , “Fluoroquinolone Antibiotics: Prescribe Only as Last Resort, Says UK Regulator,” BMJ 384 (2024): q183, 10.1136/bmj.q183. 38267066

[16] Z. Zhang , J. Lu , Y. Wang , Y. Pang , and Y. Zhao , “Prevalence and Molecular Characterization of Fluoroquinolone‐Resistant *Mycobacterium tuberculosis* Isolates in China,” Antimicrobial Agents and Chemotherapy 58 (2014): 364–369, 10.1128/AAC.01228-13. 24165186 PMC3910797

[17] A. J. Mehlhorn and D. A. Brown , “Infectious Diseases: Safety Concerns With Fluoroquinolones,” Annals of Pharmacotherapy 41 (2007): 1859–1866. 17911203 10.1345/aph.1K347

[18] S. B. Park , D. Goldstein , A. V. Krishnan , et al., “Chemotherapy‐Induced Peripheral Neurotoxicity: A Critical Analysis,” CA: A Cancer Journal for Clinicians 63 (2013): 419–437, 10.3322/caac.21204. 24590861

[19] C. S. Chen , M. P. Dorsch , S. Alsomairy , et al., “Remote Monitoring of Chemotherapy‐Induced Peripheral Neuropathy by the NeuroDetect iOS App: Observational Cohort Study of Patients With Cancer,” Journal of Medical Internet Research 27 (2025): e65615, 10.2196/65615. 39908091 PMC11840369

[20] B. R. Meyers and P. Wilkinson , “Clinical Pharmacokinetics of Antibacterial Drugs in the Elderly. Implications for Selection and Dosage,” Clinical Pharmacokinetics 17 (1989): 385–395, 10.2165/00003088-198917060-00003. 2689039

[21] J. W. Mold , S. K. Vesely , B. A. Keyl , J. B. Schenk , and M. Roberts , “The Prevalence, Predictors, and Consequences of Peripheral Sensory Neuropathy in Older Patients,” Journal of the American Board of Family Practice 17 (2004): 309–318, 10.3122/jabfm.17.5.309. 15355943

[22] Y. Li , L. Chen , X. Tang , L. Luo , and C. Wang , “Safety Analysis of Fluoroquinolone Drugs in Elderly Patients Over 65 Based on FAERS,” Expert Opinion on Drug Safety 24 (2024): 1–13, 10.1080/14740338.2024.2392862. 39269701

[23] L. D. Wu , L. D. Y. Wu , S. A. N. Walker , et al., “Antibiotic Use and Need for Antimicrobial Stewardship in Long‐Term Care,” Canadian Journal of Hospital Pharmacy 68 (2015): 445–449, 10.4212/cjhp.v68i6.1500. 26715780 PMC4690669

[24] M. L. Moro and C. Gagliotti , “Antimicrobial Resistance and Stewardship in Long‐Term Care Settings,” Future Microbiology 8 (2013): 1011–1025, 10.2217/fmb.13.75. 23902147

[25] J. Elliott , G. Sloan , L. Stevens , et al., “Female Sex Is a Risk Factor for Painful Diabetic Peripheral Neuropathy: The EURODIAB Prospective Diabetes Complications Study,” Diabetologia 67 (2024): 190–198, 10.1007/s00125-023-06025-z. 37870649 PMC10709240

[26] A. Ammendola , D. Gemini , S. Iannaccone , et al., “Gender and Peripheral Neuropathy in Chronic Alcoholism: A Clinical‐Electroneurographic Study,” Alcohol and Alcoholism 35 (2000): 368–371, 10.1093/alcalc/35.4.368. 10906002

[27] T. Miyamoto , S. Hiramoto , A. Kanto , et al., “Estrogen Decline Is a Risk Factor for Paclitaxel‐Induced Peripheral Neuropathy: Clinical Evidence Supported by a Preclinical Study,” Journal of Pharmacological Sciences 146 (2021): 49–57, 10.1016/j.jphs.2021.03.001. 33858655

[28] X. Chen , Y. Gan , N. P. B. Au , and C. H. E. Ma , “Current Understanding of the Molecular Mechanisms of Chemotherapy‐Induced Peripheral Neuropathy,” Frontiers in Molecular Neuroscience 17 (2024): 1345811, 10.3389/fnmol.2024.1345811. 38660386 PMC11039947

[29] V. B. Chine , N. P. B. Au , G. Kumar , and C. H. E. Ma , “Targeting Axon Integrity to Prevent Chemotherapy‐Induced Peripheral Neuropathy,” Molecular Neurobiology 56 (2019): 3244–3259, 10.1007/s12035-018-1301-8. 30117103

[30] V. B. Chine , N. P. B. Au , and C. H. E. Ma , “Therapeutic Benefits of Maintaining Mitochondrial Integrity and Calcium Homeostasis by Forced Expression of Hsp27 in Chemotherapy‐Induced Peripheral Neuropathy,” Neurobiology of Disease 130 (2019): 104492, 10.1016/j.nbd.2019.104492. 31176721

[31] P. Rawla , M. L. El Helou , and A. R. Vellipuram , “Fluoroquinolones and the Risk of Aortic Aneurysm or Aortic Dissection: A Systematic Review and Meta‐Analysis,” Cardiovascular & Hematological Agents in Medicinal Chemistry 17 (2019): 3–10, 10.2174/1871525717666190402121958. 30947680 PMC6865049

[32] P. P. Majalekar and P. J. Shirote , “Fluoroquinolones: Blessings or Curses,” Current Drug Targets 21 (2020): 1354–1370, 10.2174/1389450121666200621193355. 32564750

[33] M. A. Jackson , G. E. Schutze , and Diseases, C.o.I , “The Use of Systemic and Topical Fluoroquinolones,” Pediatrics 138 (2016): 459. 10.1542/peds.2016-2706 27940800

[34] J. Solomkin , E. Hershberger , B. Miller , et al., “Ceftolozane/Tazobactam Plus Metronidazole for Complicated Intra‐Abdominal Infections in an Era of Multidrug Resistance: Results From a Randomized, Double‐Blind, Phase 3 Trial (ASPECT‐cIAI),” Clinical Infectious Diseases 60 (2015): 1462–1471, 10.1093/cid/civ097. 25670823 PMC4412191

[35] S. Kalghatgi , C. S. Spina , J. C. Costello , et al., “Bactericidal Antibiotics Induce Mitochondrial Dysfunction and Oxidative Damage in Mammalian Cells,” Science Translational Medicine 5 (2013): 192ra185, 10.1126/scitranslmed.3006055. PMC3760005 23825301

[36] W. C. Tsai , C. C. Hsu , C. P. C. Chen , et al., “Ciprofloxacin Up‐Regulates Tendon Cells to Express Matrix Metalloproteinase‐2 With Degradation of Type I Collagen,” Journal of Orthopaedic Research 29 (2011): 67–73, 10.1002/jor.21196. 20602464

[37] E. Unseld , G. Ziegler , A. Gemeinhardt , U. Janssen , and U. Klotz , “Possible Interaction of Fluoroquinolones With the Benzodiazepine‐GABAA‐Receptor Complex,” British Journal of Clinical Pharmacology 30 (1990): 63–70, 10.1111/j.1365-2125.1990.tb03744.x. 2167717 PMC1368276

[38] N. P. Au , Y. Fang , N. Xi , K. W. Lai , and C. H. Ma , “Probing for Chemotherapy‐Induced Peripheral Neuropathy in Live Dorsal Root Ganglion Neurons With Atomic Force Microscopy,” Nanomedicine 10 (2014): 1323–1333, 10.1016/j.nano.2014.03.002. 24632247

[39] N. P. Au , N. P. B. Au , G. Kumar , et al., “Ciguatoxin Reduces Regenerative Capacity of Axotomized Peripheral Neurons and Delays Functional Recovery in Pre‐Exposed Mice After Peripheral Nerve Injury,” Scientific Reports 6 (2016): 26809, 10.1038/srep26809. 27229176 PMC4882531

[40] N. P. B. Au , Y. Gan , X. Chen , F. Gao , C. K. S. Leung , and C. H. E. Ma , “Transcription Factor Activator Protein 4 (AP4)‐Mediated Intrinsic Control of Axon Regeneration,” Science Advances 11 (2025): eadu3917, 10.1126/sciadv.adu3917. 41160685 PMC12571079

[41] W. Renthal , A. Chamessian , M. Curatolo , et al., “Human Cells and Networks of Pain: Transforming Pain Target Identification and Therapeutic Development,” Neuron 109 (2021): 1426–1429, 10.1016/j.neuron.2021.04.005. 33957072 PMC9208579

[42] N. P. B. Au , R. Chand , G. Kumar , et al., “A Small Molecule M1 Promotes Optic Nerve Regeneration to Restore Target‐Specific Neural Activity and Visual Function,” Proceedings of the National Academy of Sciences of the United States of America 119 (2022): e2121273119, 10.1073/pnas.2121273119. 36306327 PMC9636930

[43] I. Smyrnias and N. P. B. Au , “Proactively Restore Visual Function: Directly Targeting Affected Retinal Neurons,” Neural Regeneration Research 21 (2025): 2960–2961, 10.4103/NRR.NRR-D-25-00860. 41169218 PMC13378894

[44] A. Okifuji and B. D. Hare , “The Association Between Chronic Pain and Obesity,” Journal of Pain Research 10 (2015): 399–408. 10.2147/JPR.S55598 PMC4508090 26203274

[45] S. Tesfaye and D. Selvarajah , “Advances in the Epidemiology, Pathogenesis and Management of Diabetic Peripheral Neuropathy,” Diabetes/Metabolism Research and Reviews 28 (2012): 8–14. 10.1002/dmrr.2239 22271716

[46] T. Julian , M. Rekatsina , F. Shafique , and P. Zis , “Human Immunodeficiency Virus–Related Peripheral Neuropathy: A Systematic Review and Meta‐Analysis,” European Journal of Neurology 28 (2021): 1420–1431. 33226721 10.1111/ene.14656

[47] C. Kramarz , E. Murphy , M. M. Reilly , and A. M. Rossor , “Nutritional Peripheral Neuropathies,” Journal of Neurology, Neurosurgery & Psychiatry 95 (2024): 61–72. 10.1136/jnnp-2022-329849 37536924

[48] N. P. B. Au , G. Kumar , P. Asthana , et al., “Clinically Relevant Small‐Molecule Promotes Nerve Repair and Visual Function Recovery,” NPJ Regenerative Medicine 7 (2022): 50, 10.1038/s41536-022-00233-8. 36182946 PMC9526721

[49] N. P. B. Au , T. Wu , X. Chen , et al., “Genome‐Wide Study Reveals Novel Roles for Formin‐2 in Axon Regeneration as a Microtubule Dynamics Regulator and Therapeutic Target for Nerve Repair,” Neuron 111 (2023): 3970–3987, 10.1016/j.neuron.2023.11.011. 38086376

[50] E. A. van Niekerk , C. M. de Freria , B. O. Mancarci , et al., “Thiorphan Reprograms Neurons to Promote Functional Recovery After Spinal Cord Injury,” Nature 648 (2025): 402–408, 10.1038/s41586-025-09647-y. 41162703 PMC12695623

