# Supplementary material for: Disproportionality Analysis of Fluoroquinolone‐Associated Peripheral Neuropathy in the FAERS Database (2007–2024)
Source: Clin Transl Sci. 2026 Apr 14;19(4):e70541. doi: 10.1111/cts.70541 (PMC13079070; doi:10.1111/cts.70541)
Supplement: Supplementary file 1 — Figure S1: Flow chart illustrating the selection process of fluoroquinolone (FQ)‐associated adverse event reports (AERs). The complete dataset, comprising AERs in which any of six FDA‐approved FQs (ciprofloxacin, levofloxacin, moxifloxacin, ofloxacin, gemifloxacin, and delafloxacin) were listed as suspect drugs, was extracted from the FDA Adverse Event Reporting System (FAERS) Public Dashboard (2007–2024 Q3). Following deduplication and exclusion of AERs involving concomitant medications, a total of 37,778 cases were retained for descriptive and disproportionality analyses. Among these, 5588 cases presented with peripheral neuropathy (PN)‐related adverse events (AEs). Figure S2: Demographic characteristics of fluoroquinolone (FQ)‐associated adverse event reports (AERs) from the FAERS database (2007–2024 Q3). Proportional bar plots depicting distributions of (A) age group, (B) sex, (C) reporter type, (D) country of origin, (E) seriousness classification, and (F) clinical outcomes across all six FDA‐approved fluoroquinolones (CFX, ciprofloxacin; Com, combination therapy; DFX, delafloxacin; GFX, gemifloxacin; LFX, levofloxacin; MFX, moxifloxacin; OFX, ofloxacin). Figure S3: Demographic characteristics of FQ‐associated AERs with peripheral neuropathy (PN)‐related adverse events (AEs) from the FAERS database (2007–2024 Q3). Proportional bar plots demonstrating distributions of (A) age group, (B) sex, (C) reporter type, (D)country of origin, (E) seriousness classification, and (F) clinical outcomes for all six FDA approved FQs with PN‐related AEs (CFX, ciprofloxacin; Com, combination therapy; DFX, delafloxacin; GFX, gemifloxacin; LFX, levofloxacin; MFX, moxifloxacin; OFX, ofloxacin). (G) Clinical indications for FQ usage in these FQ‐associated AERs with PN‐related AEs are presented in descending order of frequency, with bacterial infections appeared as the majority of prescribing patterns. Table S1: Fluoroquinolones (FQs) investigated in the current study. This table pres [file CTS-19-e70541-s005.pdf]

Supplementary Materials for

Disproportionality analysis of fluoroquinolone-associated peripheral neuropathy in the FAERS database (2007-2024)

Nimra Shamim[1,2], Kelly Doughty[1,2], Hau-Tak Chau[3], James Brown[1,2], Robert Baldock[1,2,*], Ngan Pan Bennett Au[1,4,*]

[1] School of Medicine, Pharmacy and Biomedical Sciences, Faculty of Science and Health, University of Portsmouth, Portsmouth PO1 2DT, UK.

[2] Institute of Life Sciences and Healthcare, University of Portsmouth, Portsmouth PO1 2DT, UK.

[3] Department of Medicine, School of Clinical Medicine, The University of Hong Kong, Hong Kong SAR.

[4] Department of Comparative Biomedical Sciences, School of Veterinary Medicine, University of Surrey, Guildford GU2 7AL, UK.

* **Correspondence:** Dr. Ngan Pan Bennett Au

[4] Department of Comparative Biomedical Sciences, School of Veterinary Medicine, University of Surrey, Guildford GU2 7AL, UK.

Email: n.au@surrey.ac.uk

* **Correspondence:** Dr. Robert Baldock

[1] School of Medicine, Pharmacy and Biomedical Sciences, Faculty of Science and Health, University of Portsmouth, Portsmouth PO1 2DT, UK. [2] Institute of Life Sciences and Healthcare, University of Portsmouth, Portsmouth PO1 2DT, UK.

Email: robert.baldock@port.ac.uk

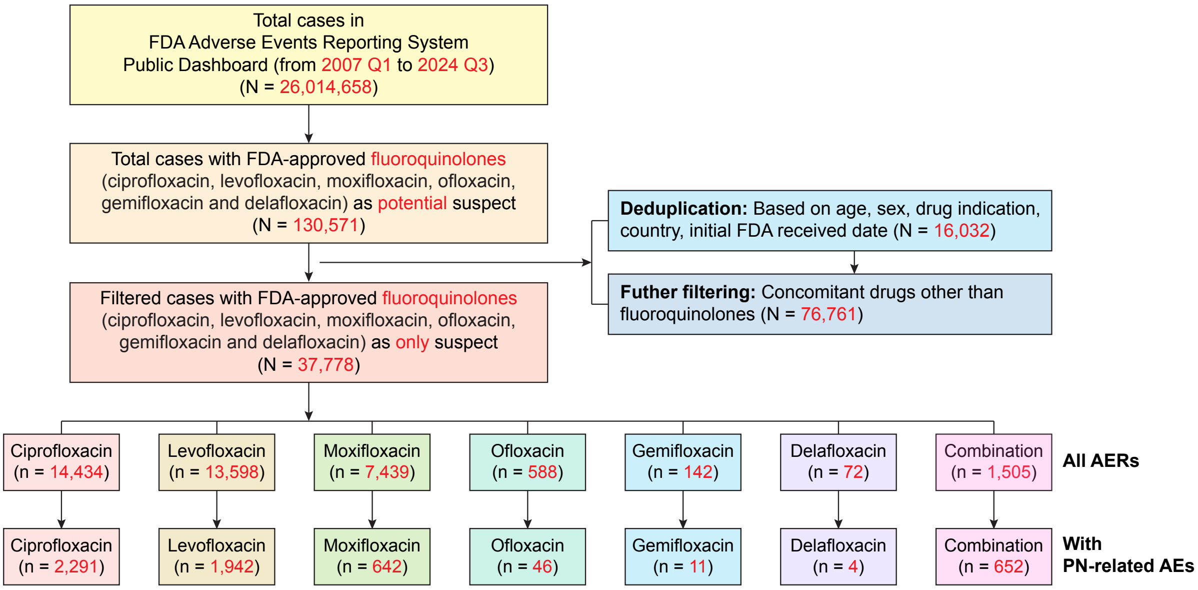

Fig. S1. Flow chart illustrating the selection process of fluoroquinolone (FQ)-associated adverse event reports (AERs). The complete dataset, comprising AERs in which any of six FDA-approved FQs (ciprofloxacin, levofloxacin, moxifloxacin, ofloxacin, gemifloxacin, and delafloxacin) were listed as suspect drugs, was extracted from the FDA Adverse Event Reporting System (FAERS) Public Dashboard (2007–2024 Q3). Following deduplication and exclusion of AERs involving concomitant medications, a total of 37,778 cases were retained for descriptive and disproportionality analyses. Among these, 5,588 cases presented with peripheral neuropathy (PN)-related adverse events (AEs).

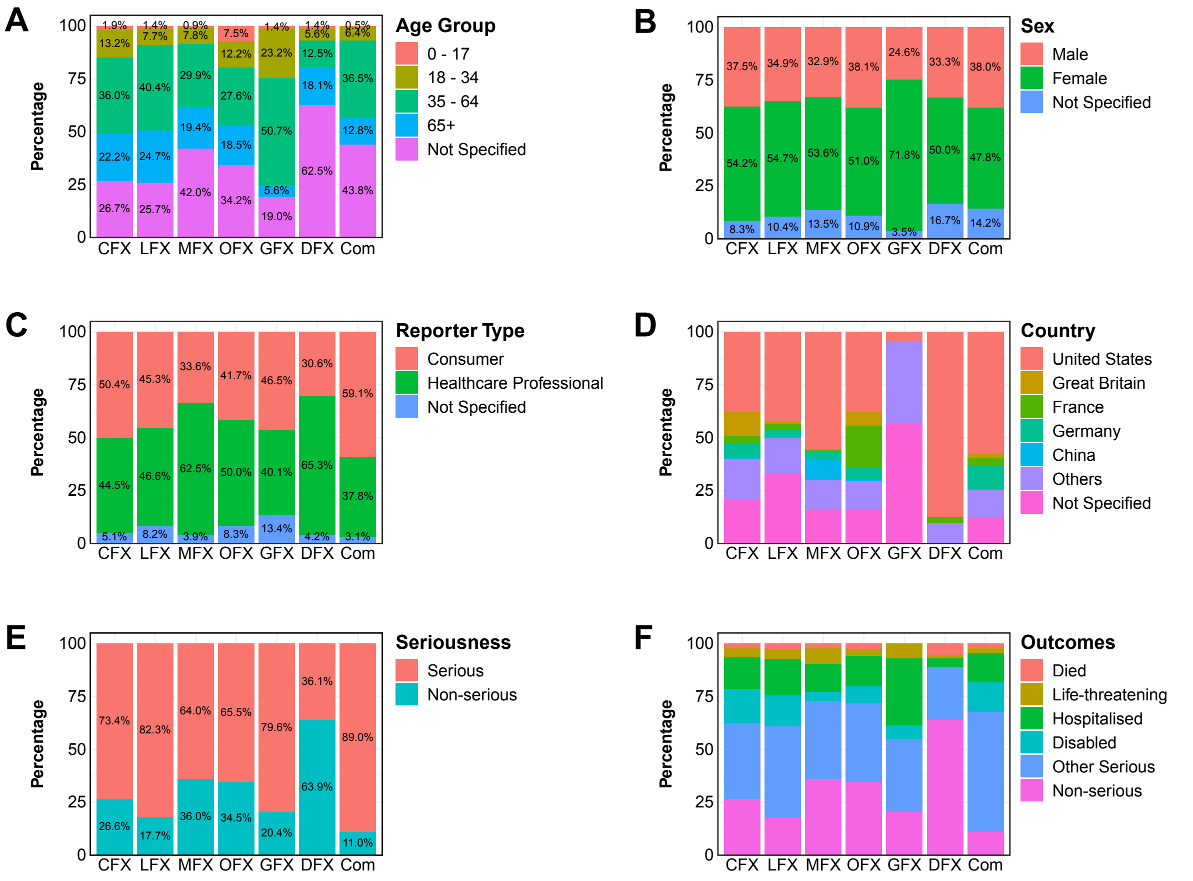

Fig. S2. Demographic characteristics of fluoroquinolone (FQ)-associated adverse event reports (AERs) from the FAERS database (2007–2024 Q3). Proportional bar plots depicting distributions of **(A)** age group, **(B)** sex, **(C)** reporter type, **(D)** country of origin, **(E)** seriousness classification, and **(F)** clinical outcomes across all six FDA-approved fluoroquinolones (CFX: ciprofloxacin; LFX: levofloxacin; MFX: moxifloxacin; OFX: ofloxacin; GFX: gemifloxacin; DFX: delafloxacin; Com: combination therapy).

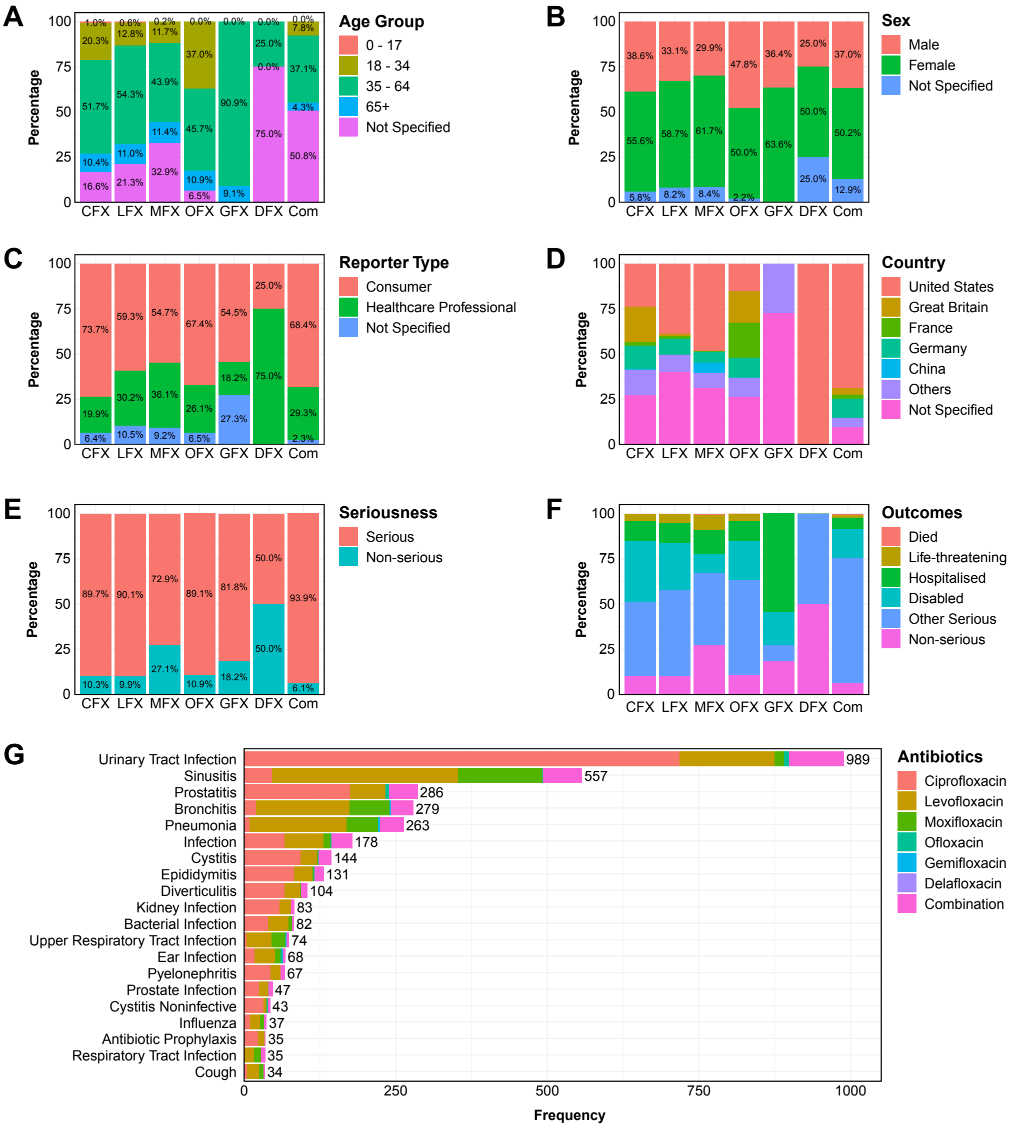

Fig. S3. Demographic characteristics of FQ-associated AERs with peripheral neuropathy (PN)-related adverse events (AEs) from the FAERS database (2007–2024 Q3). Proportional bar plots demonstrating distributions of **(A)** age group, **(B)** sex, **(C)** reporter type, **(D)** country of origin, **(E)** seriousness classification, and **(F)** clinical outcomes for all six FDA-approved FQs with PN-related AEs (CFX: ciprofloxacin; LFX: levofloxacin; MFX: moxifloxacin; OFX: ofloxacin; GFX: gemifloxacin; DFX: delafloxacin; Com: combination therapy). **(G)** Clinical indications for FQ usage in these FQ-associated AERs with PN-related AEs are presented in descending order of frequency, with bacterial infections appeared as the majority of prescribing patterns.

Table S1. Fluoroquinolones (FQs) investigated in the current study. This table presents the approval status of each FQ by the FDA, EMA and MHRA, alongside the nomenclature used for enquiries from the FAERS Public Dashboard.

Table S2. Total prescriptions of the most commonly used FQs in the United States from 2013 to 2022. Of the six FDA-approved FQs, four (ciprofloxacin, levofloxacin, moxifloxacin and ofloxacin) featured in the Top 300 Drug List, representing the most commonly prescribed FQs in the US. Data were extracted from the ClinCalc DrugStats Database (version 2024.08). All medication utilisation data originated from the annual Medical Expenditure Panel Survey (MEPS), conducted by the Agency for Healthcare Research and Quality (AHRQ) under the US government.

Table S3. Neurological characteristics of FQ-associated adverse event reports (AERs).

Supplementary Table S4. Comprehensive list of clinical indications for FQ use in FQ-associated AERs with peripheral neuropathy (PN)-related adverse events (AEs).

Table S5. Demographic characteristics of FQ-associated AERs. Data derived from FAERS database, 2007–2024 Q3.

Table S6. Demographic characteristics of FQ-associated AERs with PN-related AEs. Data derived from FAERS, 2007–2024 Q3.

Table S7. Case counts for each PN-related preferred term (PT) by individual FQs.

Supplementary Table S8. Disproportionality analysis of PN-related PTs by individual FQs.

Table S9. Classification for PN-related preferred terms (PTs).

Table S10. Univariate Logistic regression analysis of the odds ratio for FQ-associated PN-related AEs.

Table S11. Case counts of FQ-associated AERs with fatal outcomes.

Table S12. Clinical characteristics of FQ-associated PN-related AERs with fatal outcomes.

Table S1. Fluoroquinolones (FQs) investigated in the current study. This table includes the approval status of each FQ in FDA, EMA and MHRA, and names for enquiry of FAERS Publich Dashboard.

Drugs	FDA Approval Year	EMA Approval	MHRA Approval	Suspected Active Ingredients
Ciprofloxacin	1987	Yes	Yes	Ciprofloxacin, Ciprofloxacin Hydrochloride, Ciprofloxacin Lactate
Levofloxacin	1996	Yes	Yes	Levofloxacin, Levofloxacin Hemihydrate, Levofloxacin Hydrochloride, Levofloxacin Lactate, Levofloxacin Mesylate, Levofloxacin Sodium
Moxifloxacin	1999	Yes	Yes	Moxifloxacin, Moxifloxacin Hydrochloride
Ofloxacin	1990	Yes	Yes	Ofloxacin, Ofloxacin Hydrochloride
Gemifloxacin	2003	No	No	Gemifloxacin, Gemifloxacin Mesylate
Delafloxacin	2017	Yes	Yes	Delafloxacin, Delafloxacin Meglumine

Table S2. Total prescriptions of the most commonly used FQs in the United States from 2013 to 2022. Of the six FDA-approved FQs, four (ciprofloxacin, levofloxacin, moxifloxacin and ofloxacin) featured in the Top 300 Drug List, representing the most commonly prescribed FQs in the US. Data were extracted from the ClinCalc DrugStats Database (version 2024.08). All medication utilisation data originated from the annual Medical Expenditure Panel Survey (MEPS), conducted by the Agency for Healthcare Research and Quality (AHRQ) under the US government.

	Total Prescriptions in the United States Per Year (2013-2022)			
**Year**	**Ciprofloxacin**	**Levofloxacin**	**Moxifloxacin**	**Ofloxacin**
2013	7,189,978	4,323,485	1,245,912	1,299,215
2014	8,506,129	5,163,786	1,414,887	1,598,453
2015	7,793,068	4,132,454	864,455	1,307,430
2016	6,396,518	3,910,492	801,602	1,232,863
2017	6,075,065	3,777,117	537,471	1,448,901
2018	5,986,935	3,892,600	926,200	2,164,888
2019	5,878,441	3,202,649	666,288	2,051,823
2020	4,759,043	1,883,619	763,497	1,844,982
2021	4,147,677	1,889,630	713,971	1,596,736
2022	2,622,220	1,242,308	836,134	1,963,626

Table S3. Neurological characteristics of FQ-associated adverse event reports (AERs).

All AERs (2007 Q1 - 2024 Q3) **26,014,658**

	Ciprofloxacin	Levofloxacin	Moxifloxacin	Ofloxacin	Gemifloxacin	Delafloxacin	Combination	Total	*P*-value
Total AERs	14,434	13,598	7,439	588	142	72	1,505	37,778	N/A
PN-related AERs	2,291 (41.0%)	1,942 (34.8%)	642 (11.5%)	46 (0.8%)	11 (0.2%)	4 (0.1%)	652 (11.7%)	5,588 (100%)	N/A
Ratio of Neurological Reactions									
With Neurological AEs	4,584 (31.8%)	4,150 (30.5%)	2,035 (27.4%)	123 (20.9%)	30 (21.1%)	6 (8.3%)	840 (55.8%)	11,768 (31.2%)	$P < 0.0001$
Without Neurological AEs	9,850 (68.2%)	9,448 (69.5%)	5,404 (72.6%)	465 (79.1%)	112 (78.9%)	66 (91.7%)	665 (44.2%)	26,010 (68.8%)	
Ratio of PN-related AEs versus Other Neurological Reactions									
PN-related AEs	2,291 (50.0%)	1,942 (46.8%)	642 (31.5%)	46 (37.4%)	11 (36.7%)	4 (66.7%)	652 (77.6%)	5,588 (47.5%)	$P < 0.0001$
Other Neurological AEs	2,293 (50.0%)	2,208 (53.2%)	1,393 (68.5%)	77 (62.6%)	19 (63.3%)	2 (33.3%)	188 (22.4%)	6,180 (52.5%)	
Ratio of PN-related AEs versus Other AEs									
PN-related AEs	2,291 (15.9%)	1,942 (14.3%)	642 (8.6%)	46 (7.8%)	11 (7.7%)	4 (5.6%)	652 (43.3%)	5,588 (14.8%)	$P < 0.0001$
Other AEs	12,143 (84.1%)	11,656 (85.7%)	6,797 (91.4%)	542 (92.2%)	131 (92.3%)	68 (94.4%)	853 (56.7%)	32,190 (85.2%)	

Table S5. Demographic characteristics of FQ-associated AERs. Data derived from FAERS database, 2007–2024 Q3.

	Ciprofloxacin (N = 14,434)	Levofloxacin (N = 13,598)	Moxifloxacin (N = 7,439)	Ofloxacin (N = 588)	Gemifloxacin (N = 142)	Delafloxacin (N = 72)	Combination (N = 1,505)	Total (N = 37,778)	*P*-value
Age Group									*P* < 0.0001
0-17	273 (1.89%)	186 (1.37%)	70 (0.94%)	44 (7.48%)	2 (1.41%)	1 (1.39%)	7 (0.47%)	583 (1.54%)	
18-34	1,904 (13.19%)	1,049 (7.71%)	578 (7.77%)	72 (12.24%)	33 (23.24%)	4 (5.56%)	97 (6.45%)	3,737 (9.89%)	
35-64	5,199 (36.02%)	5,500 (40.45%)	2,225 (29.91%)	162 (27.55%)	72 (50.70%)	9 (12.50%)	549 (36.48%)	13,716 (36.31%)	
65+	3,208 (22.23%)	3,364 (24.74%)	1,441 (19.37%)	109 (18.54%)	8 (5.63%)	13 (18.06%)	193 (12.82%)	8,336 (22.07%)	
Not Specified	3,850 (26.67%)	3,499 (25.73%)	3,125 (42.01%)	201 (34.18%)	27 (19.01%)	45 (62.50%)	659 (43.79%)	11,406 (30.19%)	
Sex									1.04E-40
Male	5,417 (37.53%)	4,745 (34.89%)	2,449 (32.92%)	224 (38.10%)	35 (24.65%)	24 (33.33%)	572 (38.01%)	13,466 (35.65%)	
Female	7,820 (54.18%)	7,438 (54.70%)	3,989 (53.62%)	300 (51.02%)	102 (71.83%)	36 (50.00%)	719 (47.77%)	20,404 (54.01%)	
Not Specified	1,197 (8.29%)	1,415 (10.41%)	1,001 (13.46%)	64 (10.88%)	5 (3.52%)	12 (16.67%)	214 (14.22%)	3,908 (10.34%)	
Reporter Type									*P* < 0.0001
Consumer	7,271 (50.37%)	6,155 (45.26%)	2,499 (33.59%)	245 (41.67%)	66 (46.48%)	22 (30.56%)	889 (59.07%)	17,147 (45.39%)	
Healthcare Professional	6,427 (44.53%)	6,332 (46.57%)	4,649 (62.49%)	294 (50.00%)	57 (40.14%)	47 (65.28%)	569 (37.81%)	18,375 (48.64%)	
Not Specified	736 (5.10%)	1,111 (8.17%)	291 (3.91%)	49 (8.33%)	19 (13.38%)	3 (4.17%)	47 (3.12%)	2,255 (5.97%)	
Country									*P* < 0.0001
United States	5,459 (37.82%)	5,703 (41.94%)	4,133 (55.56%)	222 (37.76%)	6 (4.23%)	63 (87.50%)	860 (57.14%)	16,446 (43.53%)	
Great Britain	1,668 (11.56%)	202 (1.49%)	27 (0.36%)	39 (6.63%)	0 (0.00%)	0 (0.00%)	36 (2.39%)	1,972 (5.22%)	
France	438 (3.03%)	383 (2.82%)	65 (0.87%)	115 (19.56%)	0 (0.00%)	2 (2.78%)	55 (3.65%)	1,058 (2.80%)	
Germany	1,052 (7.29%)	494 (3.63%)	255 (3.43%)	36 (6.12%)	0 (0.00%)	0 (0.00%)	165 (10.96%)	2,002 (5.30%)	
China	24 (0.17%)	30 (0.22%)	736 (9.89%)	3 (0.51%)	0 (0.00%)	0 (0.00%)	6 (0.40%)	799 (2.11%)	
Others	2,786 (19.30%)	2,275 (16.73%)	1,015 (13.64%)	77 (13.10%)	55 (38.73%)	7 (9.72%)	195 (12.96%)	6,410 (16.97%)	
Not Specified	3,007 (20.83%)	4,511 (33.17%)	1,208 (16.24%)	96 (16.33%)	81 (57.04%)	0 (0.00%)	188 (12.49%)	9,091 (24.06%)	
Seriousness									4.84E-241
Serious	10,590 (73.37%)	11,186 (82.26%)	4,764 (64.04%)	385 (65.48%)	113 (79.58%)	26 (36.11%)	1,340 (89.04%)	28,404 (75.19%)	
Non-serious	3,844 (26.63%)	2,412 (17.74%)	2,675 (35.96%)	203 (34.52%)	29 (20.42%)	46 (63.89%)	165 (10.96%)	9,374 (24.81%)	
Outcomes									*P* < 0.0001
Died	332 (2.30%)	342 (2.52%)	171 (2.30%)	16 (2.72%)	0 (0.00%)	4 (5.56%)	33 (2.19%)	898 (2.38%)	
Life-threatening	609 (4.22%)	645 (4.74%)	539 (7.25%)	18 (3.06%)	10 (7.04%)	1 (1.39%)	39 (2.59%)	1,861 (4.93%)	
Hospitalised	2,164 (14.99%)	2,365 (17.39%)	1,002 (13.47%)	84 (14.29%)	45 (31.69%)	3 (4.17%)	204 (13.55%)	5,867 (15.53%)	
Disabled	2,360 (16.35%)	1,942 (14.28%)	299 (4.02%)	49 (8.33%)	9 (6.34%)	0 (0.00%)	212 (14.09%)	4,871 (12.89%)	
Other Serious	5,125 (35.51%)	5,892 (43.33%)	2,753 (37.01%)	218 (37.07%)	49 (34.51%)	18 (25.00%)	852 (56.61%)	14,907 (39.46%)	
Non-serious	3,844 (26.63%)	2,412 (17.74%)	2,675 (35.96%)	203 (34.52%)	29 (20.42%)	46 (63.89%)	165 (10.96%)	9,374 (24.81%)	

Table S6. Demographic characteristics of FQ-associated AERs with PN-related AEs. Data derived from FAERS, 2007–2024 Q3.

	Ciprofloxacin (N = 2,291)	Levofloxacin (N = 1,942)	Moxifloxacin (N = 642)	Ofloxacin (N = 46)	Gemifloxacin (N = 11)	Delafloxacin (N = 4)	Combinational (N = 652)	Total (N = 5,588)	*P*-value
Age Group									*P* < 0.0001
0-17	22 (0.96%)	12 (0.62%)	1 (0.16%)	0 (0.00%)	0 (0.00%)	0 (0.00%)	0 (0.00%)	35 (0.63%)	
18-34	466 (20.34%)	249 (12.82%)	75 (11.68%)	17 (36.96%)	0 (0.00%)	0 (0.00%)	51 (7.82%)	858 (15.35%)	
35-64	1,184 (51.68%)	1,055 (54.33%)	282 (43.93%)	21 (45.65%)	10 (90.91%)	1 (25.00%)	242 (37.12%)	2,795 (50.02%)	
65+	238 (10.39%)	213 (10.97%)	73 (11.37%)	5 (10.87%)	1 (9.09%)	0 (0.00%)	28 (4.29%)	558 (9.99%)	
Not Specified	381 (16.63%)	413 (21.27%)	211 (32.87%)	3 (6.52%)	0 (0.00%)	3 (75.00%)	331 (50.77%)	1,342 (24.02%)	
Sex									*P* < 0.0001
Male	885 (38.63%)	643 (33.11%)	192 (29.91%)	22 (47.83%)	4 (36.36%)	1 (25.00%)	241 (36.96%)	1,988 (35.58%)	
Female	1,273 (55.57%)	1,140 (58.70%)	396 (61.68%)	23 (50.00%)	7 (63.64%)	2 (50.00%)	327 (50.15%)	3,168 (56.69%)	
Not Specified	133 (5.81%)	159 (8.19%)	54 (8.41%)	1 (2.17%)	0 (0.00%)	1 (25.00%)	84 (12.88%)	432 (7.73%)	
Reporter Type									*P* < 0.0001
Consumer	1,689 (73.72%)	1,151 (59.27%)	351 (54.67%)	31 (67.39%)	6 (54.55%)	1 (25.00%)	446 (68.40%)	3,675 (65.77%)	
Healthcare Professional	455 (19.86%)	587 (30.23%)	232 (36.14%)	12 (26.09%)	2 (18.18%)	3 (75.00%)	191 (29.29%)	1,482 (26.52%)	
Not Specified	147 (6.42%)	204 (10.50%)	59 (9.19%)	3 (6.52%)	3 (27.27%)	0 (0.00%)	15 (2.30%)	431 (7.71%)	
Country									*P* < 0.0001
United States	549 (23.96%)	753 (38.77%)	310 (48.29%)	7 (15.22%)	0 (0.00%)	4 (100.00%)	449 (68.87%)	2,072 (37.08%)	
Great Britain	446 (19.47%)	28 (1.44%)	1 (0.16%)	8 (17.39%)	0 (0.00%)	0 (0.00%)	24 (3.68%)	507 (9.07%)	
France	47 (2.05%)	24 (1.24%)	2 (0.31%)	9 (19.57%)	0 (0.00%)	0 (0.00%)	14 (2.15%)	96 (1.72%)	
Germany	300 (13.09%)	172 (8.86%)	38 (5.92%)	5 (10.87%)	0 (0.00%)	0 (0.00%)	70 (10.74%)	585 (10.47%)	
China	1 (0.04%)	3 (0.15%)	37 (5.76%)	0 (0.00%)	0 (0.00%)	0 (0.00%)	0 (0.00%)	41 (0.73%)	
Others	323 (14.10%)	187 (9.63%)	55 (8.57%)	5 (10.87%)	3 (27.27%)	0 (0.00%)	33 (5.06%)	606 (10.84%)	
Not Specified	625 (27.28%)	775 (39.91%)	199 (31.00%)	12 (26.09%)	8 (72.73%)	0 (0.00%)	62 (9.51%)	1,681 (30.08%)	
Seriousness									*P* < 0.0001
Serious	2,056 (89.74%)	1,750 (90.11%)	468 (72.90%)	41 (89.13%)	9 (81.82%)	2 (50.00%)	612 (93.87%)	4,938 (88.37%)	
Non-serious	235 (10.26%)	192 (9.89%)	174 (27.10%)	5 (10.87%)	2 (18.18%)	2 (50.00%)	40 (6.13%)	650 (11.63%)	
Outcomes									*P* < 0.0001
Died	9 (0.39%)	8 (0.41%)	3 (0.47%)	0 (0.00%)	0 (0.00%)	0 (0.00%)	5 (0.77%)	25 (0.45%)	
Life-threatening	91 (3.97%)	98 (5.05%)	55 (8.57%)	2 (4.35%)	0 (0.00%)	0 (0.00%)	10 (1.53%)	256 (4.58%)	
Hospitalised	255 (11.13%)	212 (10.92%)	84 (13.08%)	5 (10.87%)	6 (54.55%)	0 (0.00%)	42 (6.44%)	604 (10.81%)	
Disabled	768 (33.52%)	497 (25.59%)	70 (10.90%)	10 (21.74%)	2 (18.18%)	0 (0.00%)	105 (16.10%)	1,452 (25.98%)	
Other Serious	933 (40.72%)	935 (48.15%)	256 (39.88%)	24 (52.17%)	1 (9.09%)	2 (50.00%)	450 (69.02%)	2,601 (46.55%)	
Non-serious	235 (10.26%)	192 (9.89%)	174 (27.10%)	5 (10.87%)	2 (18.18%)	2 (50.00%)	40 (6.13%)	650 (11.63%)	

Table S7. Case counts for each PN-related preferred term (PT) by individual FQs.

PN-related Clinical Indications	Ciprofloxacin (N = 2,291)	Levofloxacin (N = 1,942)	Moxifloxacin (N = 642)	Ofloxacin (N = 46)	Gemifloxacin (N = 11)	Delafloxacin (N = 4)	Combinational (N = 652)	Total (N = 5,588)
Paraesthesia	1,039	662	276	26	4	2	86	2,095
Neuropathy Peripheral	668	683	131	8	0	2	494	1,986
Hypoaesthesia	760	520	231	15	4	2	105	1,637
Burning Sensation	485	361	140	8	5	1	66	1,066
Sensory Disturbance	95	60	15	3	0	0	15	188
Polyneuropathy	69	54	1	1	0	0	10	135
Small Fibre Neuropathy	23	30	7	0	0	0	26	86
Hyperaesthesia	36	32	5	3	0	0	5	81
Sensory Loss	33	24	7	1	0	0	1	66
Dysaesthesia	34	12	3	0	0	0	5	54
Unresponsive To Stimuli	7	19	18	0	1	0	4	49
Electric Shock Sensation	32	14	2	0	0	0	0	48
Peripheral Sensory Neuropathy	15	16	1	1	0	0	5	38
Peripheral Sensorimotor Neuropathy	3	13	0	0	0	0	5	21
Allodynia	13	6	0	0	0	0	0	19
Axonal Neuropathy	8	3	0	0	0	0	5	16
Peripheral Motor Neuropathy	3	7	0	0	0	0	1	11
Demyelinating Polyneuropathy	2	7	0	0	0	0	1	10
Toxic Neuropathy	3	3	0	0	0	0	1	7
Acute Polyneuropathy	1	5	0	0	0	0	0	6
Hyperpathia	5	0	0	0	0	0	0	5
Slow Response To Stimuli	0	1	3	0	0	0	0	4
Mononeuropathy	0	2	0	0	0	0	1	3
Sciatic Nerve Neuropathy	1	1	1	0	0	0	0	3
Acute Motor Axonal Neuropathy	2	0	0	0	0	0	0	2
Acute Motor-Sensory Axonal Neuropathy	1	0	0	0	0	0	0	1
Hyporesponsive To Stimuli	0	1	0	0	0	0	0	1
Subacute Inflammatory Demyelinating Polyneuropathy	1	0	0	0	0	0	0	1

Table S9. Classification for PN-related preferred terms (PTs).

Category	Perferred Term
Sensory Hyposensitivity	Hypoaesthesia
	Sensory Loss
	Unresponsive To Stimuli
	Hyporesponsive To Stimuli
	Slow Response To Stimuli
Sensory Hypersensitivity	Paraesthesia
	Burning Sensation
	Electric Shock Sensation
	Dysaesthesia
	Hyperaesthesia
	Allodynia
	Hyperpathia
Unclassified Sensory Symptoms	Neuropathy Peripheral
	Sensory Disturbance
	Polyneuropathy
	Small Fibre Neuropathy
	Axonal Neuropathy
	Demyelinating Polyneuropathy
	Toxic Neuropathy
	Acute Polyneuropathy
	Sciatic Nerve Neuropathy
	Peripheral Sensory Neuropathy
	Acute Motor-Sensory Axonal Neuropathy
	Mononeuropathy
	Peripheral Sensorimotor Neuropathy
Motor Symptoms	Peripheral Motor Neuropathy
	Acute Motor-Sensory Axonal Neuropathy
	Acute Motor Axonal Neuropathy
	Subacute Inflammatory Demyelinating Polyneuropathy
	Peripheral Sensorimotor Neuropathy

Table S10. Univariate Logistic regression analysis of the odds ratio for FQ-associated PN-related AEs.

Variable	Factor	Odds Ratio	Lower Limit of 95% CI	Upper Limit of 95% CI	*P*-value
Antibiotics	Ciprofloxacin	1	-	-	-
	Levofloxacin	0.89	0.84	0.96	1.73E-02
	Moxifloxacin	0.53	0.48	0.58	4.23E-38
	Ofloxacin	0.51	0.37	0.69	2.78E-04
	Gemifloxacin	0.36	0.18	0.63	1.40E-02
	Delafloxacin	0.51	0.15	1.24	1
	Combination	3.74	3.33	4.20	1.52E-107
Sex	Male	1	-	-	-
	Female	1.12	1.05	1.19	6.20E-03
Age Group	0-17	1	-	-	-
	18-34	3.93	2.83	5.63	7.85E-14
	35-64	3.32	2.41	4.74	4.00E-11
	65+	0.94	0.67	1.35	1
Seriousness	Non-serious	1	-	-	-
	Serious	1.97	1.80	2.16	1.18E-46
	Critical	3.08	2.79	3.40	7.08E-107

Table S11. Case counts of FQ-associated AERs with fatal outcomes.

	With PN AEs (N = 5,588)	Without PN AEs (N = 32,190)	Total (N = 37,778)	*P*-value
Non-fatal	5,563 (99.55%)	31,317 (97.29%)	36,880 (97.62%)	1.78E-24
Fatal	25 (0.45%)	873 (2.71%)	898 (2.38%)	

Table S12. Clinical characteristics of FQ-associated PN-related AERs with fatal outcomes.

	Non-fatal (N = 36,880)	Fatal (N = 898)	Total (N = 37,778)	*P*-value
Age Group				3.47E-29
0-17	569 (1.54%)	14 (1.56%)	583 (1.54%)	
18-34	3,671 (9.95%)	66 (7.35%)	3,737 (9.89%)	
35-64	13,512 (36.64%)	204 (22.72%)	13,716 (36.31%)	
65+	8,009 (21.72%)	327 (36.41%)	8,336 (22.07%)	
Not Specified	11,119 (30.15%)	287 (31.96%)	11,406 (30.19%)	
Sex				2.52E-28
Male	13,053 (35.39%)	413 (45.99%)	13,466 (35.65%)	
Female	20,077 (54.44%)	327 (36.41%)	20,404 (54.01%)	
Not Specified	3,750 (10.17%)	158 (17.59%)	3,908 (10.34%)	
Treatment Strategy				0.69
Monotherapy	35,408 (97.62%)	865 (2.38%)	36,273 (96.02%)	
Combination	1,472 (97.81%)	33 (2.19%)	1,505 (3.98%)	
