# Supplementary material for: Disproportionality Analysis of Fluoroquinolone‐Associated Peripheral Neuropathy in the FAERS Database (2007–2024)
Source: Clin Transl Sci. 2026 Apr 14;19(4):e70541. doi: 10.1111/cts.70541 (PMC13079070; doi:10.1111/cts.70541)
Supplement: Supplementary file 4 — Data S1: The READUS‐PV checklist. [file CTS-19-e70541-s004.docx]

**The READUS-PV checklist**

| **Section and topic** | **Item #** | **Checklist item** | **Location where item is reported** |
| --- | --- | --- | --- |
| **Title** |  |  |  |
|  | *1a* | *If disproportionality analyses are a prominent component of the published study, the study should be identified as a “disproportionality analysis”. The type of data and name of the database(s) should be specified.* | Page 1, Line 1-2 |
|  | *1b* | *Report the name of adverse event(s) and/or drug(s) under study, when applicable.* | Page 1, Line 1-2 |
| **Introduction** |  |  |  |
| Background | *2a* | *Describe the drug(s) and its utilization, the nature of the adverse event(s) under study and its frequency, and the existing knowledge on the drug-event combination.* | Page 8, Line 84-91 |
|  | *2b* | *Specify the rationale for performing the analysis, e.g., as part of routine pharmacovigilance, to investigate an overall safety profile, or to assess a pre-specified hypothesis.* | Page 8, Line 92-97 |
|  | *2c* | *Explain why ICSR databases and disproportionality analysis are suitable to fill the knowledge gap.* | Page 8, Line 100-103 |
| Objectives | *3* | *State specific objectives, identifying the adverse event(s), the drug(s), and the reference group, including any pre-specified hypothesis, if applicable.* | Page 8, Line 103-106 |
| **Methods** |  |  |  |
| Study design | *4a* | *Identify the study (i.e., “disproportionality analysis”) and the type of data used (e.g., “individual case safety reports”).* | Page 7, Line 112-121; Page 8, Line 133-137 |
|  | *4b* | *Provide an outline of the entire study design, including primary and sensitivity analyses performed, and other designs such as case-by-case analysis or literature review.* | Page 7-8, Line 112-131 |
| Data description, access, and pre-processing | *5a* | *Specify the name of the database(s), the database(s) custodian, and the coverage. Specify the type/number of drugs included within the database and the thesaurus, taxonomies, or ontologies used for coding drugs and events.* | Page 7, Line 112-114; 123-131 |
|  | *5b* | *Specify the extraction dates and describe and justify all choices used for data pre-processing, including any data transformation or exclusion, if appropriate.* | Page 7, Line 123-131 |
| Variables definition | *6a* | *Describe the study population, including any restriction.* | Page 7-8, Line 123-137 |
|  | *6b* | *Describe the nature and the meaning of key variables assessed in the work.* | Page 8, Line 133-149 |
|  | *6c* | *Specify and justify any grouping of drugs or events. For drugs, specify and justify whether active ingredients/trade names/salts were considered and/or the selected role.* | Page 7, Line 112-121 |
|  | *6d* | *Describe any additional data source used, the type of data, and how they interact with ICSRs.* | Not applicable |
| Statistical methods | *7a* | *Present any descriptive analysis performed, specifying variables investigated, statistical tests, and significance thresholds.* | Page 8, Line 143-149 |
|  | *7b* | *Describe the measure(s) selected for the disproportionality analysis including any threshold used to identify signals of disproportionate reporting. Explain the reason for this choice if applicable.* | Page 8, Line 133-137 |
|  | *7c* | *Clearly describe any sensitivity analysis and any tool to control confounding, including any restriction, subgroup, stratification, adjustment, or interaction.* | Page 7-8, Line 123-149 |
|  | *7d* | *Specify the variables and methods used for the case-by-case analysis, including any algorithm or criteria used to assess causality, if performed.* | Not applicable |
|  | *7e* | *Specify any statistical methods used for other data sources.* | Page 8, Line 133-149 |
| **Results** |  |  |  |
| Participants | *8a* | *Specify the number of individual case safety reports included at each stage, including reasons for exclusion.* | Page 9, Line 153-160 |
|  | *8b* | *Provide key demographic and clinical characteristics of cases, if possible comparing cases with any appropriate reference group.* | Page 9-10, Line 161-189 |
| Disproportionality analysis | *9* | *Present all results including confidence intervals. Present also results of sensitivity analyses, if performed.* | Page 10-13, Line 192-261 |
| Case-by-case analysis | *10* | *Present the case-by-case analysis of key variables. Present the causality assessment, if applicable.* | Not feasible for FAERS database. |
| **Discussion** |  |  |  |
| Key results | *11* | *Discuss key results with reference to study objectives and contextualize them within the current literature and other consulted sources. Clearly discriminate between expected reactions and emerging safety signals.* | Page 15, Line 325-332 |
| External validity | *12a* | *Discuss the external validity of the results to the general population.* | Page 17-18, Line 352-378 |
|  | *12b* | *Discuss the potential relevance of results in clinical practice* | Page 16, Line 333-351; Page 18, 379-395 |
|  | *12c* | *Propose further study designs if applicable* | Page 20, Line 437-447 |
| Limitations | *13* | *Present general limitations, making clear that disproportionality analysis alone cannot prove causation or measure incidence, and specific limitations, including confounding and reporting bias and efforts to mitigate them.* | Page 19-20, Line 410-436 |
| **Declarations** |  |  |  |
|  | *14a* | *Provide the source of funding/sponsorship and the role of the funders/sponsors for the present study and for any original study on which the present article is based.* | Page 21, Line 453-456 |
|  | *14b* | *Clearly identify potential commercial and intellectual conflicts of interest (e.g., link to any drug/event investigated, whether financial, legal action, or software used).* | Page 21, Line 461-463 |
|  | *14c* | *Declare any institutional approval needed or granted in the investigation.* | Page 7, Line 109-110 |
|  | *14d* | *Include a statement on data availability, code availability (including the version of the statistical software used), and protocol registration.* | Page 21, Line 456-458 |
