# Supplementary material for: Disproportionality Analysis of Fluoroquinolone‐Associated Peripheral Neuropathy in the FAERS Database (2007–2024)
Source: Clin Transl Sci. 2026 Apr 14;19(4):e70541. doi: 10.1111/cts.70541 (PMC13079070; doi:10.1111/cts.70541)
Supplement: Supplementary file 5 — Data S2: The READUS‐PV checklist for abstracts. [file CTS-19-e70541-s003.docx]

**The READUS-PV checklist for abstracts**

| **Section and topic** | **Item #** | **Checklist item** | **Location where item is reported** |
| --- | --- | --- | --- |
| Background | *1a* | *State the aim/rationale for performing the study.* | Page 2, Line 25-29. |
|  | *1b* | *Specify the adverse event(s) and/or the drug(s) under study, when applicable.* | Page 2, Line 25-29. |
|  | *1c* | *Specify the specific population or setting, when applicable.* | Page 2, Line 29-32. |
| Methods | *2a* | *Identify the study as a “disproportionality analysis” and specify the type of data used.* | Page 2, Line 29-34 |
|  | *2b* | *Specify the name of the database(s) used and the type of access.* | Page 2, Line 31 |
|  | *2c* | *Specify the timeframe and geographical region, when applicable.* | Page 2, Line 31 |
|  | *2d* | *Specify the disproportionality measure(s) used and their statistical significance threshold(s).* | Page 2, Line 32-34 |
|  | *2e* | *Specify if a case-by-case analysis is performed.* | Not applicable |
| Results | *3* | *Report main findings including their precision (e.g., 95% confidence intervals), together with a short summary of the case-by-case analysis.* | Page 2, Line 34-42 |
| Conclusion | *4a* | *Clearly report key conclusions.* | Page 2, Line 42-47 |
|  | *4b* | *Acknowledge that the disproportionality analysis is a hypothesis generating or refinement approach.* | Page 2, Line 32-34 |
|  | *4c* | *State the implications and clinical relevance of the findings.* | Page 2, Line 42-47 |
